# Electrical Resonance in the θ Frequency Range in Olfactory Amygdala Neurons

**DOI:** 10.1371/journal.pone.0085826

**Published:** 2014-01-21

**Authors:** Jorge Vera, Maurizio Pezzoli, Ulises Pereira, Juan Bacigalupo, Magdalena Sanhueza

**Affiliations:** Department of Biology, Faculty of Sciences, University of Chile, Santiago, Chile; Monell Chemical Senses Center, United States of America

## Abstract

The cortical amygdala receives direct olfactory inputs and is thought to participate in processing and learning of biologically relevant olfactory cues. As for other brain structures implicated in learning, the principal neurons of the anterior cortical nucleus (ACo) exhibit intrinsic subthreshold membrane potential oscillations in the θ-frequency range. Here we show that nearly 50% of ACo layer II neurons also display electrical resonance, consisting of selective responsiveness to stimuli of a preferential frequency (2–6 Hz). Their impedance profile resembles an electrical band-pass filter with a peak at the preferred frequency, in contrast to the low-pass filter properties of other neurons. Most ACo resonant neurons displayed frequency preference along the whole subthreshold voltage range. We used pharmacological tools to identify the voltage-dependent conductances implicated in resonance. A hyperpolarization-activated cationic current depending on HCN channels underlies resonance at resting and hyperpolarized potentials; notably, this current also participates in resonance at depolarized subthreshold voltages. KV7/KCNQ K^+^ channels also contribute to resonant behavior at depolarized potentials, but not in all resonant cells. Moreover, resonance was strongly attenuated after blockade of voltage-dependent persistent Na^+^ channels, suggesting an amplifying role. Remarkably, resonant neurons presented a higher firing probability for stimuli of the preferred frequency. To fully understand the mechanisms underlying resonance in these neurons, we developed a comprehensive conductance-based model including the aforementioned and leak conductances, as well as Hodgkin and Huxley-type channels. The model reproduces the resonant impedance profile and our pharmacological results, allowing a quantitative evaluation of the contribution of each conductance to resonance. It also replicates selective spiking at the resonant frequency and allows a prediction of the temperature-dependent shift in resonance frequency. Our results provide a complete characterization of the resonant behavior of olfactory amygdala neurons and shed light on a putative mechanism for network activity coordination in the intact brain.

## Introduction

The amygdala complex is a heterogeneous group of subcortical nuclei and cortical areas located in the temporal lobe of the brain [1]. This complex is involved in the processing of biologically relevant sensory stimuli and in the generation of the autonomic, motor and endocrine responses induced by these stimuli [2], [3]. Moreover, several lines of evidence indicate that the lateral and basolateral subcortical amygdala nuclei (also known as the basolateral complex) are implicated in forms of associative learning and emotional memory, particularly in the case of fear conditioning paradigms and emotional stress [4], [5].

The wide representation and the particular organization of olfactory connections to the amygdala distinguish the olfactory system from other sensory modalities, making it a privileged model for the study of the encoding of biologically relevant stimuli and memory processes involving emotions. Surprisingly, this possibility has been scarcely explored so far. While inputs from most sensory systems enter the amygdala at the basolateral complex via the thalamus and neocortical regions, afferent connections from the main olfactory bulb (OB) directly target the amygdala at its cortical region. OB mitral and tufted cells project their axons through the lateral olfactory tract to the piriform cortex (PC) and the amygdaloid cortical nuclei (anterior cortical nucleus, ACo, and posterolateral cortical nucleus; [6]). These nuclei have a laminar configuration, with an external cell-sparse layer (layer I) that mainly contains axon collaterals from the olfactory tract and apical dendrites from the principal cells located in the more dense layer II [1]. This olfactory region has been poorly investigated, but recent anatomical evidence suggest a role in innate odor preference [7], [8]. Moreover, a behavioral and electrophysiological study supports its participation in olfactory fear conditioning in rats, as after training the synaptic potentials evoked by lateral olfactory tract stimulation are persistently potentiated specifically in ACo [7].

We previously showed that a significant fraction of principal neurons from ACo (68%) and the posterolateral cortical nucleus (20%) displays intrinsic subthreshold membrane potential oscillations (MPOs) upon depolarization by DC current injection, mainly in the θ-frequency range (3–12 Hz) [8]. Similar θ rhythmic properties have been described in neurons from brain regions implicated in learning, such as the basolateral amygdala [9], the hippocampus [10] and the entorhinal cortex (EC) [11].

In addition to MPOs, neurons from memory-related brain regions like the hippocampus, the EC and the lateral amygdala display θ-frequency subthreshold resonance [9], [12], [13]. Electrical resonance is the property of certain neurons to respond with a maximal voltage signal to the injection of a fluctuating current of a specific frequency (the resonance frequency, *f_res_*), in contrast to most neurons that do not exhibit a preferred stimulation frequency, functioning mainly as low-pass filters. Intrinsic pacemaker properties and resonance rely on voltage-dependent conductances [14], [15]. Resonance is generated by voltage-dependent currents that dynamically oppose to voltage changes, with long activation/deactivation times relative to the membrane time constant [15]. For fluctuating stimuli with frequencies low enough to allow the activation of these currents, the voltage response will be attenuated. The coexistence of this high-pass filter mechanism and the low-pass filter generated by the membrane passive properties gives rise to the band-pass filtering that defines resonance. Two specific active currents that have been shown to participate in θ resonance are the hyperpolarization-activated K^+^/Na^+^ current *I_h_* [16] and the slow outward rectifier K^+^ current regulated by muscarinic receptors, *I_m_* [17], [18]. Subthreshold resonance depending on either *I_h_* or *I_m_* has been reported in different regions of the rodent brain. *I_h_*–dependent resonance is observed in rat subiculum and EC [19]–[21]. On the other hand, a resonance mechanism relying on *I_m_* exists in frontal cortex neurons of guinea pig [22], but not of rat, where it is mediated by *I_h_* [23]. Similarly, different reports claim for the involvement of either *I_h_* or *I_m_* in basolateral amygdala θ-resonance [24], [25]. Finally, a dual mechanism exists in rat CA1 pyramidal neurons, where resonance at hyperpolarized potentials relies on *I_h_* and at depolarized voltages, on *I_m_* [26].

In resonant neurons, subthreshold frequency preference may selectively translate to spiking patterns incoming oscillatory inputs at the resonance frequency. It could thus constitute a band-pass filter mechanism for transmitting repetitive activity in a limited frequency range, which may critically contribute to orchestrate neuronal network rhythms [14], [15]. Neuronal resonance in the θ-frequency range may participate in the generation of the network θ waves arising during memory formation, suggesting an involvement of this intrinsic property in learning. Accordingly, during network θ activity the induction of synaptic plasticity is facilitated [27].

The mammalian olfactory circuit is characterized by a pronounced θ-frequency activity, which is behaviorally driven by sniffing [28]. At rest, breathing frequency is around 2–4 Hz, while sniffing frequency during exploration and odor recognition tasks is around 6–12 Hz, thus rhythmic activity spans the whole θ range [29]–[31]. Sniffing evokes phase-locked spiking in principal cells of the OB and PC [32]–[34] and correlated rhythmic activity in the hippocampus, a correlation that increases when the animal is evaluating the biological significance of the stimulus [35]. In this context, it becomes relevant to assess the existence of θ resonance in neurons from the olfactory amygdala, a possible locus of olfactory-related emotional learning.

To assess neuronal resonance in ACo, we used the standard impedance amplitude profile (ZAP) protocol [15], [36], which consists in the injection of a sinusoidal current of constant amplitude and linearly changing frequency. By impedance analysis we were able to identify layer II ACo resonant neurons and determine their resonance frequencies. We identified the conductances involved in resonance generation at different voltage ranges and developed a comprehensive computational model that reproduces both subthreshold resonance and neuronal spiking behavior during ZAP stimulation.

## Materials and Methods

### Ethical approval

Animal care and experimental procedures were approved by the Bio-Ethical Committee of the Faculty of Sciences, University of Chile, according to the ethical rules of the Biosafety Policy Manual of the National Fund for Scientific and Technological Development (FONDECYT).

### Slice preparation

Male Sprague Dawley rats from 18 to 30 day-old were used. The animals were deeply anesthetized with ether and sacrificed by decapitation. The brain was rapidly removed and transferred to an ice-cold dissection solution containing (in mM): 213 sucrose, 2.6 KCl, 10 MgCl_2_, 0.5 CaCl_2_, 26 NaHCO_3_, 1.3 NaH_2_PO_4_ and 10 dextrose (equilibrated with 95% O_2_ and 5% CO_2_), pH 7.3. Coronal slices (400 µm) containing the ACo (Bregma −2.2 to −3.3; Paxinos et al., 1999) were obtained with a vibratome (Vibratome Sectioning System 102, Pelco). The slices were placed in a holding chamber with artificial cerebro-spinal fluid (ACSF) containing (in mM): 125 NaCl, 2.5 KCl, 1.25 NaH_2_PO_4_, 25 NaHCO_3_, 10 Glucose, 1 MgCl_2_, 2 CaCl_2_ (equilibrated with 95% O_2_ and 5% CO_2_), pH 7.3. The slices were left to recover during at least 1 h at 30°C before using them for recordings.

### Electrophysiological recordings

Whole cell patch-clamp recordings were conducted under visual guidance by an upright microscope equipped with oblique infrared optics (Olympus BX51WI). Electrodes (3.5–5 MΩ) were fabricated from borosilicate glass capillary tubing (0.8–1.10×100 mm; Kimble Glass Inc) using a horizontal puller (Flaming/Brown P- 97, Sutter Instrument Co). Current-clamp recordings were made with an EPC-9 patch-clamp amplifier (Heka, Heidelberg, Germany), data were filtered at 16 kHz and acquired at 1 or 25 kHz using the Heka Pulse software. Patch pipettes were filled with internal solution containing (in mM): 130 K-gluconate, 5 KCl, 2 MgCl_2_, 0.6 EGTA, 10 HEPES, 4 Mg-ATP, 0.3 Na_3_-GTP (pH 7.3, 280 mOsm). Membrane voltages reported here were corrected for liquid junction potential (∼12 mV, measured according to standard procedure [37]). The input resistance of the neurons was calculated by measuring the voltage deflections at the end of hyperpolarizing current pulses of 10–30 pA and 200 ms duration. Experiments were conducted at 28–30°C and the recording chamber was continuously perfused with oxygenated ACSF (2–3 ml/min). Only neurons with resting membrane potential more negative than −60 mV were included in this study. On-line analysis was carried out using Heka Pulse software.

### ZAP stimulation and analysis

Voltage responses to an intracellularly injected pseudo-sinusoidal current of constant amplitude (10 pA) and linearly decreasing or increasing frequencies (ZAP stimuli; frequency interval: 0–15 or 20 Hz, 10 s duration) were recorded in current clamp conditions. The full stimulation protocol included a complete screening of the physiologically relevant subthreshold membrane potentials. For this, ZAP stimuli were superposed to a series of incremental 10 pA current steps of 11 s duration, from about −50 pA until action potentials were triggered. In experiments with blockers of voltage-dependent channels the amplitude of ZAP stimuli was adjusted to maintain a peak to peak subthreshold voltage response comparable to the control condition (5–10 mV) and to favor the evaluation of perithreshold resonance in the absence of spikes. In all experiment the protocol was repeated 8 to 10 times in every neuron, for each condition. The output isopotential subthreshold waves were averaged to proceed with the impedance analysis.

The impedance frequency profile (*Z(f)*) was obtained from the ratio of Fast Fourier Transforms (FFT) of output (voltage) and input (current) waves (*Z(f) = FFT[V(t)]/FFT[I(t)*]), using Igor Pro software version 5.01 (Wavemetrics, Inc., Lake Oswego, OR). The impedance is a complex quantity (*Z(f)  =  Z, Real + iZ, Imaginary*), where the real part (*Z, Real*) is the resistance and the imaginary part (*Z, Imaginary*), the reactance. For each given frequency, the complex impedance can be plotted as a vector whose magnitude and phase (*φ_z_(f)*; angle with the real axis) are, respectively given by the following expressions: 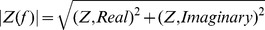
(1)

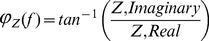
(2)


Throughout the text the term *impedance* will be used to refer to the magnitude of the impedance vector, unless otherwise stated. The impedance phase corresponds to the phase shift of the voltage wave relative to the current wave. Frequencies below 0.5 Hz were not plotted in the graphs for impedance and phase profiles, to avoid low frequency distortions. In some pharmacological experiments, the impedance profiles were normalized to the value at the maximal frequency, to allow easier discrimination of changes in curve shape from overall shifts that may occur after manipulations that modify membrane resistance. Off-line analyses and graphs were performed with Igor Pro or Microsoft Excel programs. Average results are expressed as mean ± SD. Student's-t test set at a level p<0.05 was used as criterion of significance.

### Quantification of resonance

Resonance is defined as the band-pass filter property of the impedance profile [15]. The strength of resonance is usually quantified as the ratio between the maximal impedance (i.e. the impedance at the resonance frequency, *|Z(f_res_)|*) and the impedance at the lowest frequency (|*Z(0.5)*|). This ratio is called the Q factor or value and indicates the sharpness of the impedance curve around the resonance frequency. For a more precise determination of Q, the experimental data were fitted with a theoretical curve for the impedance [38], obtained from the resolution of a phenomenological linearized membrane circuit model for resonance, in which the band-pass filter properties result from the addition of an inductive (L) branch to the electric circuit that models the passive membrane properties (RC circuit, consisting of a resistance and a capacitor in parallel, reproducing the low-pass membrane filtering) [15], [39]. The impedance of an RLC circuit has band-pass (resonating) properties and a characteristic phase profile that is different from the RC case (see examples and discussion below). The inductive branch that generates the high-pass filter component results from the influence of voltage-dependent currents called inductive currents. Here we chose Q≥1.10 as a quantitative criterion to differentiate resonant from non-resonant cells, thus the maximal impedance should be at least 10% higher than |*Z(0.5)*|. We set this criterion to guarantee that even for noisy impedance profiles, the average of 10 points around the peak were statistically different from the average of the same number of points at the lowest frequency. For simplicity, this was considered as a general cutoff criterion for resonance in this paper and its accuracy is discussed in Results.

### Drugs

Drugs were bath applied at the following final concentrations: 10 µM 6-cyano-7-nitoquinoxaline-2,3-dione (CNQX; AMPA-type glutamate receptor antagonist), 100 µM d-2-amino-5- phosphonovaleric acid (APV; NMDA-type glutamate receptor antagonist), 100 µM picrotoxin (PTX; GABA-A receptor blocker), 1 µM tetrodotoxin (TTX; voltage-dependent Na^+^ channel blocker), 10 µM XE991 (KCNQ channel blocker), 4.0 mM CsCl. Drugs were obtained from Sigma, except for XE991 and ZD7288, purchased to Tocris, and TTX, that was obtained from Alomone Labs. The synaptic blockers were present in the pharmacological experiments performed to identify ionic currents involved in resonance.

### Conductance-based model and computer simulations

Following the Hodgkin-Huxley formalism [40], we developed a comprehensive conductance-based single compartment model. The model included a passive leak current (*I_leak_*), a hyperpolarization-activated cation current, *I_h_* [41], a persistent (non-inactivating) Na^+^ current (*I_NaP_*) [42] and a slow muscarine-regulated K^+^ current (*I_m_*) [43], as well as modified Hodgkin-Huxley- type fast Na^+^ and delayed rectifier K^+^ currents (*I_Na,H_* and *I_K,H,_* respectively) [40]. The equation describing the evolution of membrane voltage (V) with time is 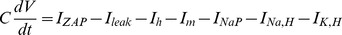
(3)where *C* is the membrane capacitance and *I_ZAP_* is the applied current. The first four transmembrane ionic currents in Eq. 3 follow the set of equations [44]: 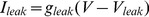
(4)

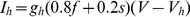
(5)

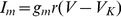
(6)

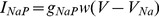
(7)with g*_leak_*, *g_h_*, g*_m_* and *g_NaP_* being the maximal conductances of the corresponding currents and *V_leak_*, *V_h_*, *V_K_* and *V_Na_* the reversal potentials of *I_leak_*, *I_h_*, K^+^- and Na^+^- mediated currents, respectively. Finally, the dynamics of the state variables 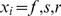 and 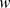 is ruled by the following equation: 
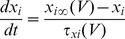
(8)where 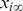 are the steady-state values of 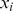, and 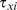 are the corresponding time constants. A summary of the reversal potentials and the equations ruling steady-state variables and time constants for the different currents is shown in Table 1. Voltage-dependence of state variables and time constants for *I_h_* were taken from [41], and for *I_m_* and *I_NaP_*, from [40]. The τ values for *I_h_* where divided by the temperature-correcting factor 4.5^(T-38)/10^ [45] and those for *I_m_*, by the factor 3^(T-22)/10^ [40]. Unless something else is stated, the temperature was set at 30°C to mimic experimental conditions.

**Table 1:** Parameters and equations used for calculation of ionic currents.

Current	Reversal Potential (mV)	State Variables at equilibrium	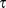 (ms)
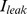	−71	-	-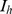	−25	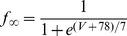	38
		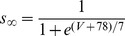	319
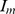	−100	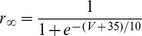	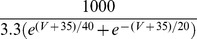
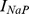	125	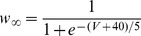	5

On the other hand, the equations describing the Hodgkin-Huxley Na^+^ and K^+^ currents are: 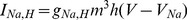
(9)

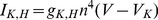
(10)where *g_K,H_* and *g_Na,H_* are the maximal conductances and 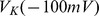 and 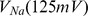 are the equilibrium potentials. The dynamics of the state variables *m, h* and *n* also follows Eq. 8, but in this case the equilibrium values 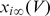 are given by the equation: 
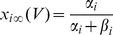
(11)


The rate constants α_i_ and β_i_ were calculated following a set of modified Hodgkin-Huxley equations for cortical neurons [44] and are listed in Table 2. All the expressions in Table 2 where multiplied by the temperature correcting factor 10^(T-6.3)/10^ [44].

**Table 2:** Equations used for calculation of rate constants α_i_ and β_i_.

	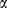	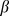
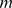	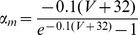	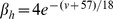
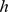	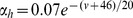	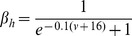
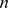	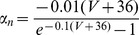	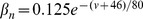

Simulations were performed using NEURON 7.0 environment [46] on a Macintosh computer. A cylindrical single compartment model (28 µm length, 28 µm diameter and a capacitance density of 1 µFcm^−2^) was set to account for the capacitance of olfactory amygdala resonant neurons. An integration time step of 25 µs (40 kHz) was used for all simulations. The axial resistivity R_a_ of the compartment was set to 35.4 Ωcm and the membrane input resistance R_i_ was set according to experimentally measured values (see Results). We explored the other parameters of the model to find the membrane potential dynamics that mimicked the electrophysiological results. To simulate pharmacological experiments, the maximal conductance of the channels targeted by each drug was set to zero. The stimulation protocol used in all the cases comprised at least 5 ZAP current injections, each of 10 s duration and 10 pA amplitude, and ranging from 15–0 Hz. The 5 ZAP injections were superposed to increasing current steps that moved the average potential between approximately −85 mV to −60 mV, as for the electrophysiological experiments. The code for reproducing the computer simulations described in this paper is available in the model database ModelDB (http://senselab.med.yale.edu/modeldb/).

## Results

Whole-cell current-clamp recordings were performed in 156 layer II neurons of the anterior cortical nucleus of the amygdala (ACo). Only cells with resting membrane potential (V_r_) more negative than −60 mV were considered in this study. In addition, recordings from 86 cells were conducted in the presence of blockers of fast glutamatergic and GABA-ergic synaptic transmission (10 µM CNQX, 100 µM AP5, 100 µM picrotoxin).

### Resonant and non-resonant neurons in ACo

**Figure 1:**
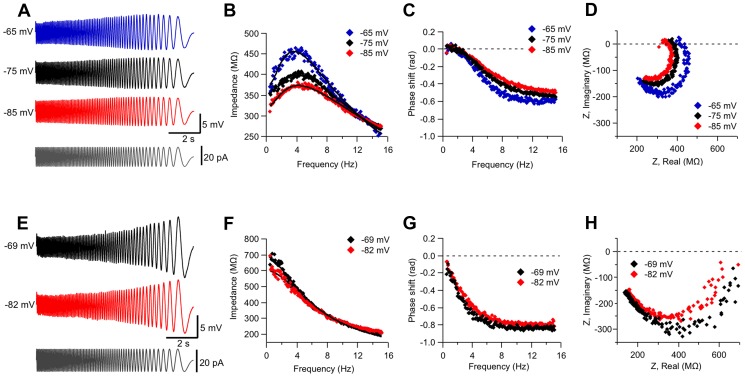
Resonant and non-resonant layer II ACo neurons. *A*–*D*, Voltage waveforms and impedance analysis from a representative resonant neuron. *A*, Voltage response to a 10 s ZAP stimulus (lower trace) of 10 pA amplitude and linearly decreasing frequency (15–0 Hz), at different membrane potentials. *B*, Resonant impedance profiles for data shown in A. Data were fitted by a theoretical curve obtained from the phenomenological RLC circuit model (lines; Q = 1.22, 1.12, 1.15 and *f_res_* = 3.6, 4.0, 4.2 Hz, for −65, −75 and −85 mV, respectively). *C*, Phase shift of the voltage waves relative to the injected current waves, as a function of frequency. *D*, Impedance vectors are represented as points in the complex plane. Here the distance to the origin corresponds to the impedance magnitude (plotted in B) and the angle with the real axis represents the phase shift or impedance phase (shown in C). Frequency increases in the clockwise direction. *E*–*H*, Same as A–D, for a non-resonant cell.

Inspection of neuronal output voltage waves to ZAP stimulation and their impedance profiles (see Methods) suggested the existence of two populations of ACo layer II cells with different subthreshold behaviors: resonant and non-resonant. Figures 1A–D and 1E–H show voltage waves and impedance analysis for two ACo neurons exemplifying resonant and non-resonant types, respectively. Panels 1A and 1E illustrate the voltage responses to ZAP stimuli applied superposed to DC current injections of different amplitudes. In the non-resonant cell, the maximal voltage response was attained at the lowest stimulus frequency (Fig.1E), as expected from general passive RC membrane properties acting as a low-pass filter (see impedance profiles in Figure 1F). In contrast, for the cell in 1A the highest voltage amplitude was reached at a narrow frequency interval within the range scanned by the ZAP stimulus (Fig. 1A), giving rise to band-pass filter profiles (Fig. 1B) that are indicative of resonant behavior. This suggests the existence in this neuron of voltage-dependent currents that decrease the ZAP-induced voltage fluctuations at low frequencies. Such resonant impedance profile can be described by an RLC equivalent circuit ([38]; see Methods). The degree of resonance (Q factor or value; see definition in Methods) and the resonance frequency (*f_res_*) were measured by fitting to the experimental impedance profiles a theoretical curve obtained from this linearized model. The model has the advantage of being applicable even if the specific conductances involved in the generation of the resonant profile are not known, but it has other limitations that are discussed below. Therefore, it was used here mainly as a tool to discriminate and quantify resonant behavior.

The representative resonant neuron in Figure 1A–D displays band-pass filter properties in the whole subthreshold voltage range. Least-square fits of impedance data to the theoretical curves are shown in Figure1B (black lines). The Q values for −65, −75 and −85 mV were 1.22, 1.12 and 1.15, respectively, and *f_res_* were 3.7, 4.1 and 4.2 Hz, respectively. On the other hand, the non-resonant neuron presented Q = 1.00 for all potentials recorded (only two of them are shown).

The impedance phase (or phase shift of the voltage wave with respect to the injected current wave), also has different frequency profiles for neurons described by RC (non-resonant) or RLC (resonant) electrical circuits [15], [39]. In the first case, the voltage always lags the current wave due to the membrane capacitive properties, and the phase increases monotonically with the oscillation frequency until reaching a plateau (Fig. 1G). In the second case, the slow inductive currents that oppose voltage changes at low frequencies reduce not only the amplitude of voltage deflections but also the time to reach peak values are reached, compared to the non-resonant profile. This is manifested as a reduction in the phase lag of the voltage wave relative to the injected current at low frequencies. On the other hand, at higher frequencies the capacitive component dominates (Fig. 1C; compare to Fig. 1G). At frequencies lower than *f_res_*, the inductive properties may dominate over passive low-pass filter effects, thus generating positive phase values (the voltage wave precedes the current wave; see Discussion). For the resonant cell in Figure 1C this phenomenon is observed for frequencies lower than 2 Hz, while the reduction in voltage lag is already apparent for frequencies around *f_res_* (compare with Figure 1G). The complex representation of impedance, summarizing information of its magnitude and phase (see Methods), is shown in Figures 1D and H, for the different voltages. Here the impedance magnitude corresponds to the length of the vectors connecting the origin to each point. Positive ordinate values represent positive phase shifts of the output voltage with respect to the input wave (Fig. 1D).

According to our discrimination criterion (Q≥1.10), one half of the cells recorded in regular ACSF (79 out of 156; 51%) displayed resonance in at least one sub-range of voltage (see below) and were classified as resonant neurons. We found no statistically significant differences between the passive electrical properties of resonant and non-resonant cells; average results (from 10 neurons of each type) were, respectively, R_i_ = 280±62 MΩ and 266±81 MΩ (p = 0.79), C = 60±22 pF and 79±27 pF (p = 0.09), τ = 16±5 ms and 20±6 ms (p = 0.12), and V_r_ = 67.8±4.6 mV and 65.2±5.6 mV (p = 0.33). To confirm that resonance was caused by frequency-dependent filter membrane properties instead of other time-dependent processes, we applied ZAP stimuli of increasing and decreasing frequencies in 30 cells. The impedance profiles obtained were indistinguishable in both cases (not shown). We showed that resonance was an intrinsic property of neurons, not involving network effects in its generation mechanism, as it was still observed in the presence of the inhibitors of fast glutamatergic and GABAergic synaptic transmission mentioned above (39 out of 86 cells). We did not find qualitative differences in the resonant profiles for the two conditions.

**Figure 2:**
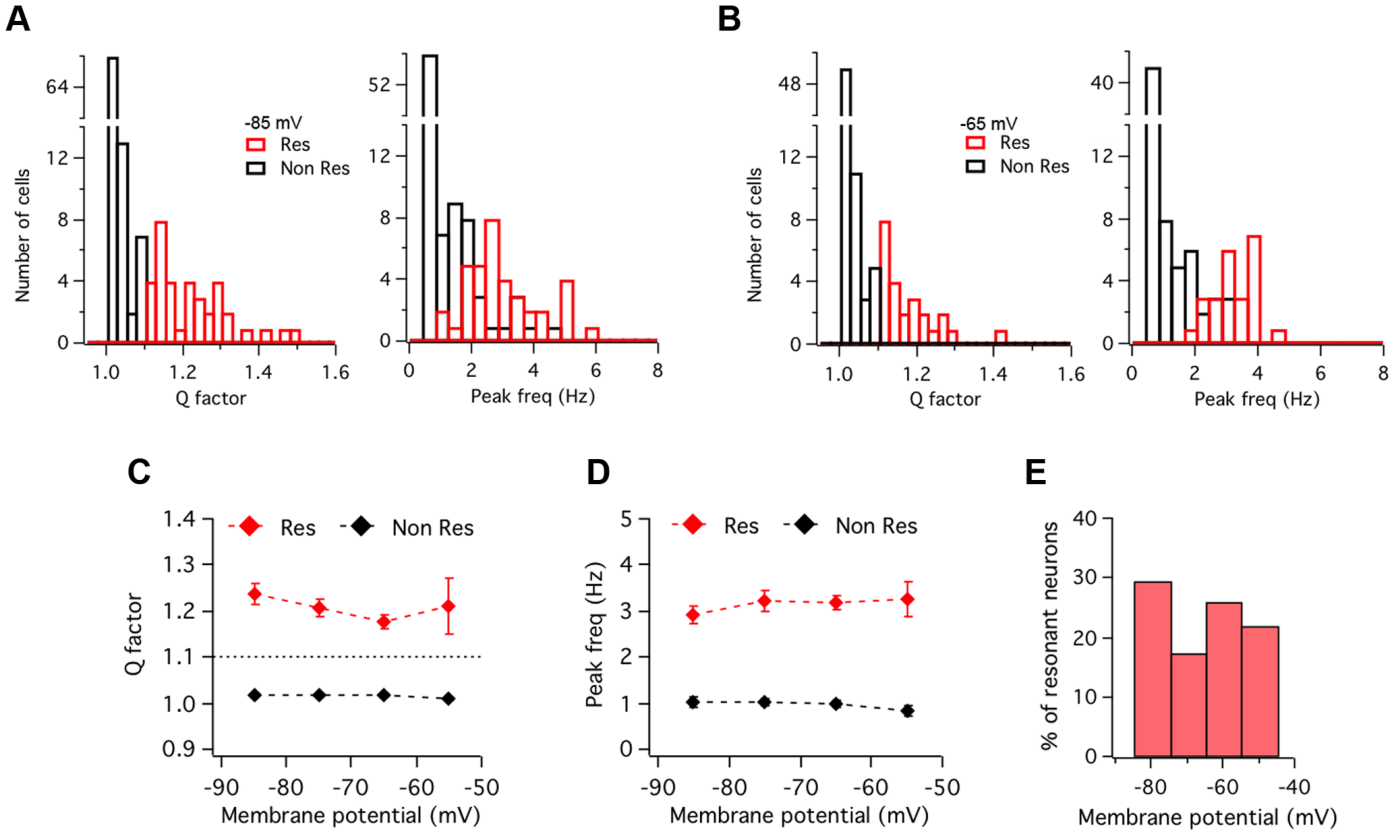
Q value and peak frequency distributions confirm the existence of resonant and non-resonant subpopulations of neurons. *A*, Histograms of the Q factors (left) and the frequencies at which Z reaches its maximum (peak frequency; right), for ZAP stimulation at an average voltage of −85 mV (i.e., in the range from −80 to −89 mV; n = 125 neurons). Data with Q<1.10 are shown in black and that with Q≥1.10, in red. *B*, Same as in A, for −65 mV (−60 to −69 mV; n = 92 neurons). *C*, Average Q factor for resonant and non-resonant ACo neurons at different membrane potentials. *D*, Average frequency at Z_max_ (peak frequency) as a function of voltage, for both subpopulations. *E*, Percentage of neurons displaying resonant behavior at the indicated voltage ranges (30, 17, 26 and 22% for −85, −75, −65 and −55 mV, respectively). The analyses in C–F were obtained from the pooled data from 156 neurons. The number of cells recorded at each potential range was 125, 132, 92 and 41, for −85, −75, −65 and −55 mV, respectively (note that not all neurons were recorded at the four potential ranges).

We observed that the majority of cells displaying resonant behavior presented this property over the whole subthreshold voltage range, as in the example shown in Figure1. However, in several cases action potentials were triggered when exploring the depolarized voltage range. In other cases, the quantitative threshold criterion for resonance was not fulfilled both below and above the resting potential, even though a trend to display band-pass filter properties was often observed, as indicated by both the voltage waveform and impedance profile (Q>1.00). Thus, instead of classifying neurons in terms of the voltages at which they did or did not display resonance we opted for a graphic representation of the voltage-dependent filter properties of the whole population of recorded neurons, including those classified as resonant and non-resonant; we made histograms of the Q values measured at different voltage ranges. The left panels of Figures 2A and B display the histograms for two voltage ranges (10 mV wide), centered at −85 and −65 mV, respectively. Histograms for all subthreshold voltage ranges were included in Figure S1 (the number of recorded cells was 125, 132, 92 and 41, for −85, −75, −65 and −55 mV, respectively). Cells displaying Q≥1.10 are shown in red and those with Q<1.10, in black. The histograms show a large subpopulation of neurons clearly falling in the category of non-resonant cells with Q identical to 1.00, which means that the maximal impedance is observed exactly at the lowest frequency (0.5 Hz). This highly populated class of neurons is followed by a group of cells with 1.00<Q<1.05, most probably belonging to the same non-resonant type as it is separated from a second clearly differentiated subpopulation of cells with Q values close to or higher than 1.10. Note that the histograms suggest that a few cases (7% and 9% of total, for −85 mV and −65 mV, respectively) in the range 1.05<Q<1.10 that were classified as non resonant by the criterion Q≥1.10, may actually belong to the resonant subpopulation. However, as our criterion was set to avoid misclassification of noisy profiles, we prefer to consider these small percentages into the experimental uncertainty instead of overestimating the proportion of resonant cells. Probably the most important outcome of the histograms shown in the left panels of Figure 2A,B, is that they confirm the existence of two distinct populations of ACo layer II cells, resonant and non-resonant, instead of just one population with different grades of resonant behavior. This conclusion is further supported by a plot of the average Q values of resonant and non-resonant cells as a function of voltage (Fig. 2C), where the two populations are clearly distinguishable. Remarkably, Figure 2C indicates that resonant behavior does not disappear at resting membrane potential, in contrast to what happens in CA1 neurons [26].

In the right panels of Figures 2A, B we plotted the frequency at which the peak impedance was observed (corresponding to *f_res_* in the case of resonant cells and shown here in red). Histograms illustrate the range of frequencies observed at these two membrane potential ranges and Figure 2D shows the average values for the different subthreshold voltage ranges. Finally, the percentage of resonant cells per membrane potential range is shown in Figure 2E. Note that these percentages (30% or less) are not inconsistent with our previous statement that about 50% of ACo neurons present resonance at least at one potential range.

In the next sections we describe a series of experiments in which pharmacological tools where used to identify the membrane conductances implicated in resonance at hyperpolarized and depolarized potentials in ACo.

### Role of the voltage-gated persistent Na^+^ current (I_NaP_)

**Figure 3:**
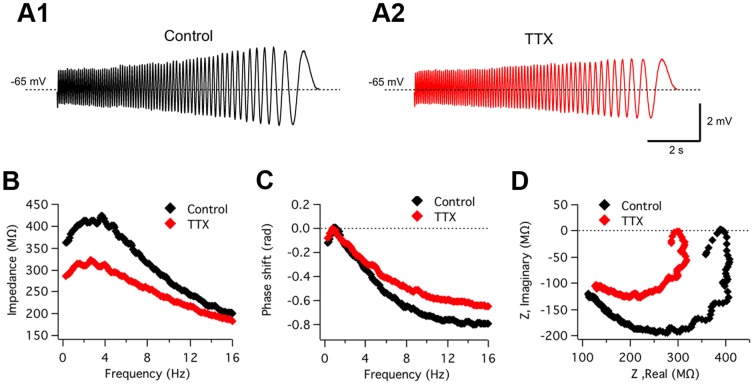
*I_NaP_* plays a critical amplifying role in resonance but is not involved in band-pass filtering. Representative experiment (n = 6) illustrating the effect on resonance of blocking voltage-dependent Na^+^ currents. *A*, Voltage responses evoked by ZAP stimulation in a resonant neuron before (A1; Control) and during (A2) the extracellular application of TTX (1 µM). *B*, Impedance profiles before (black) and during (red) TTX treatment. *f_res_* and Q value in control conditions were 3.0 Hz and 1.25, and during TTX superfusion, 2.4 Hz and 1.08, respectively. *C and D*, Comparison of phase shift profiles and complex impedance representations, respectively, indicating that while impedance amplitude was strongly reduced by TTX (see also B), the phase resonant spectrum is still present The ZAP protocol was 20–0 Hz and 10 pA amplitude. See text for average data from six experiments. ZAP stimulus: 15–0 Hz, 10 pA.

Subthreshold membrane potential oscillations and neuronal resonance are usually thought to be related phenomena involving similar voltage-dependent membrane conductances, even though the role and impact of these conductances in the two processes may not necessarily be the same [15]. As a first attempt to identify the ion channels implicated in ACo resonance, we tested if the blockade of persistent voltage-gated Na^+^ currents, that we had previously shown to abolish oscillations [8], could affect resonance. Figure 3 shows an example of a resonant neuron recorded before and during bath application of tetrodotoxin (TTX, 1 µM). Comparison of output waves (Fig. 3A1, A2) and impedance profiles (Fig. 3B) indicates that the band-pass filter properties are strongly attenuated for depolarized potentials under TTX. An overall reduction of voltage response is observed, mainly for lower frequencies. The average effect of this drug on resonance in the group of cells studied was quantified in terms of the changes in Q. On average, Q value at −65 mV was reduced from 1.14±0.04 in control conditions, to 1.03±0.04 in the presence of TTX (n = 6, p = 0.002; paired t-test). Figures 3C and D show the phase shift profile and complex impedance plots for control and TTX conditions. The plot in Figure 3C shows that in the presence of TTX the phase profile preserves the inductive properties at low frequencies that distinguish resonant from non-resonant neurons (compare with Figs. 1C and G). Instead, TTX caused an increase in phase at high frequencies (see Discussion). Figure 3D also illustrates the phase shift conservation at low frequencies (compare with Figs. 1D and H), including phase values close or above zero, while the strong TTX-induced impedance decrease is apparent. The fact that phase properties at low frequencies were preserved while resonance pattern was clearly reduced in the impedance curve, suggests that the conductances responsible for the inductive membrane properties were not targeted by the toxin. Therefore, it seems probable that, as reported for other rat brain regions [15], [26], a persistent voltage-gated Na^+^ current amplifies the resonance behavior, instead of taking part in their induction mechanism. In agreement with this interpretation, TTX application to a non-resonant neuron (Fig. S2) also reduced the impedance; this attenuation was higher for more depolarized baseline potentials, revealing a general amplifying effect not specifically related to the resonance phenomenon.

### Voltage-dependent currents involved in resonance: contribution of *I_h_* and *I_m_*

**Figure 4:**
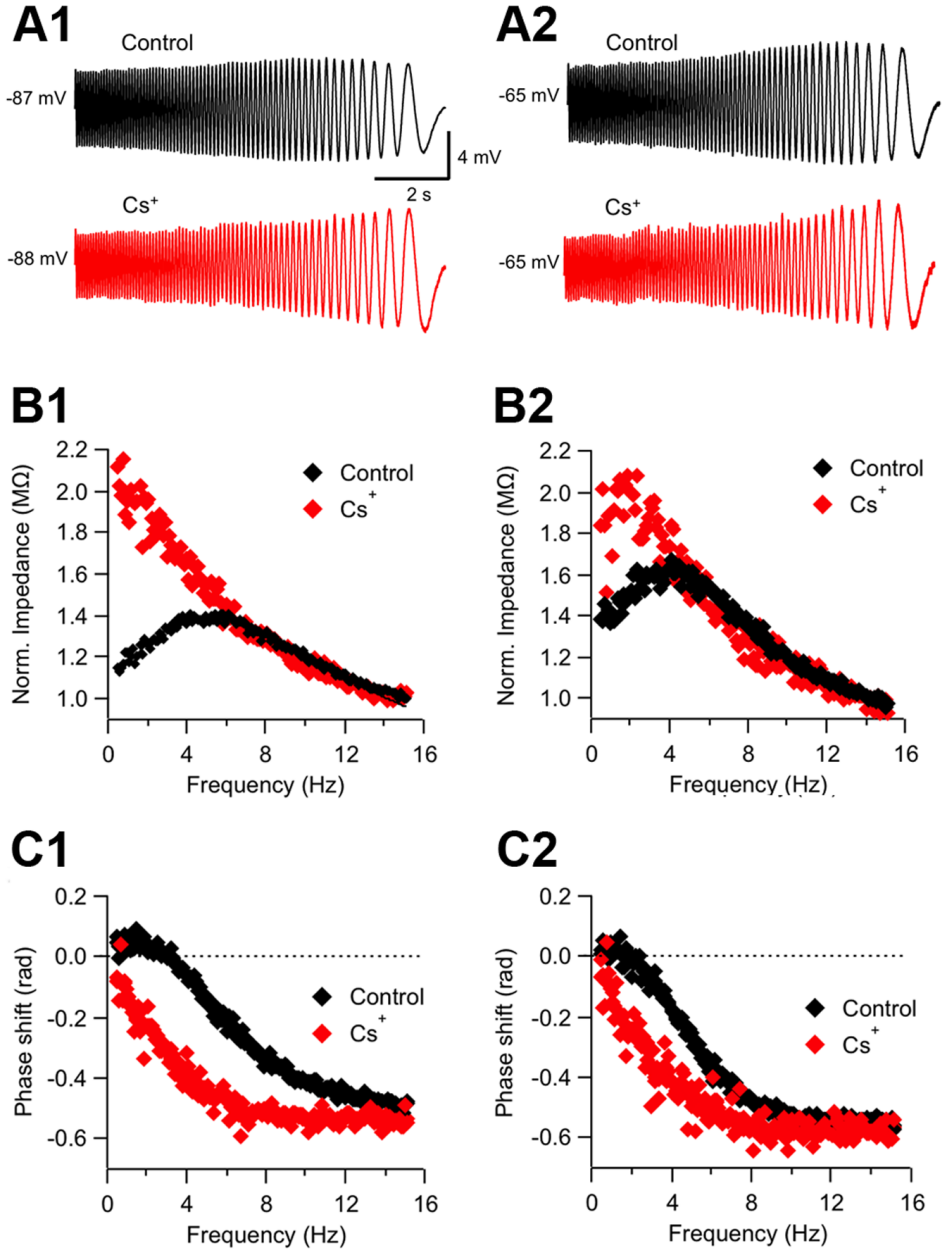
Resonance blockade by external Cs^+^. *A1*, ZAP-stimulus-induced voltage responses of a resonant neuron at a hyperpolarized potential (n = 7), before (Control) and during extracellular application of Cs^+^ (4 mM). *B1*, Normalized impedance profiles for the traces shown in A1. Note that in the presence of Cs^+^ impedance increases at low frequencies and resonance is completely lost (Q = 1.21 for Control and 1.00 for Cs^+^, as calculated from the least-squares fitted curve). *C1*, Phase shift for both conditions plotted against frequency, indicating that the inductive profile is missing in the presence of Cs^+^. *A2*–*C2*, same as A1–C1, for a depolarized potential (−65 mV; n = 4), Q = 1.19 in control conditions and 1.00 in Cs^+^. See text for average data from nine experiments. ZAP stimulus: 15–0 Hz, 10 pA.

As mentioned above, two specific active currents presenting biophysical properties consistent with a participation in θ resonance at the analyzed voltage ranges are *I_h_* and *I_m_*. We first evaluated the contribution of these currents to ACo neurons resonance by using pharmacological tools. As *I_h_* is known to be blocked by extracellular Cs^+^ in the low millimolar range [41], [47], [48], we tested if bath applications of 4 mM Cs^+^ (n = 9) affected voltage waveforms and impedance or phase profiles. An example of these experiments is shown in Figure 4. Note that here the impedance profiles before and after drug application were normalized (see Methods) for a more straightforward comparison of profile shape and to cancel any global shifts due to input resistance modification. We found that at hyperpolarized potentials resonant profiles were completely lost during Cs^+^ application (Fig. 4A1; similar results were obtained at −77 and −70 mV, not included). In contrast to TTX, Cs^+^ increased impedance in the lower frequency range (Fig. 4B1), consistent with the removal of a high-pass filter mechanism, represented by the theoretical inductive branch of the RLC phenomenological model circuit, but resulting from the action of specific voltage-dependent conductances in resonant cells. The average Q values at hyperpolarized potentials for all tested resonant cells before and after Cs^+^ application were Q = 1.17±0.09 for Control and 1.01±0.01, for Cs^+^ (p = 0.0009, n = 9; paired t-test). The phase curve was also deeply modified by the blocker (Fig. 4C1), the phase shift reduction and the positive lags originally observed for low frequencies disappeared and the curve was transformed from one resembling an RLC circuit (Fig. 1C) to a mono-phase function characteristic of an RC circuit (Figs. 1G). This result differs from the effect of TTX, as in that case the resonant phase profile was preserved (Fig. 3C). This result and the aforementioned increase in impedance indicate that, in contrast to TTX, Cs^+^ targets the resonance-generating mechanism.

In the range of the hyperpolarized potentials considered here and at resting potential, the only known current sensitive to extracellular Cs^+^ is *I_h_*. Therefore, our results suggest that this is the current responsible for resonance at these voltages. Moreover, Cs^+^ application had no effect on non-resonant cells, suggesting that *I_h_* is very small or absent in these cells (Fig. S3A1–3).

Interestingly, in the experiment shown in Figure 4 both voltage waveforms (Fig. 4A2) and resonant impedance profile (Fig. 4B2) were also deeply affected by Cs^+^ at depolarized potentials. Moreover, the resonant phase profile was also modified, being now closer to the RC behavior (Fig. 4C2). Considering all experiments with Cs^+^, in those cells in which a resonant profile was resolved at subthreshold depolarized potentials without action potential discharges (∼−65 mV; 4 cells), resonance strength showed a trend to decrease in Cs^+^, though the difference did not reach statistical significance (Q = 1.27±0.15 for Control and 1.03±0.03 for Cs^+^; p = 0.054; paired t-test). These experiments suggest a contribution of *I_h_* to resonance also at perithreshold voltages, in contrast to what has been reported for CA1 pyramidal cells, for which at depolarized potentials resonance relies exclusively on *I_m_* [26]. To assess a possible contribution of both *I_m_* and *I_h_* to perithreshold resonance in ACo neurons, we used more specific pharmacological tools.

**Figure 5:**
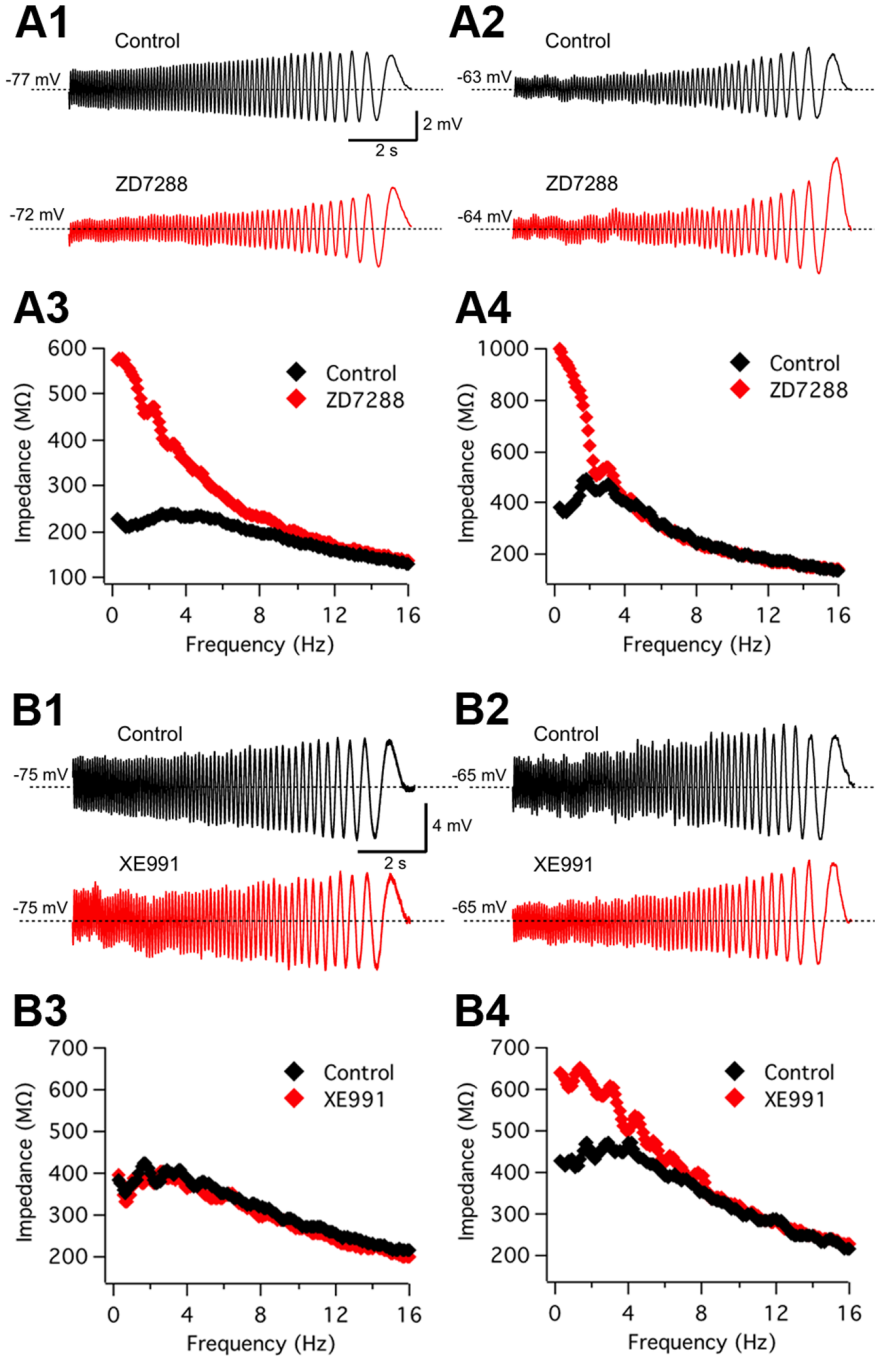
Subthreshold resonance depends on *I_h_* in the whole subthreshold range; *I_m_* also contributes in a subset of neurons at depolarized potentials. *A1*–*A4*, Effect on resonance of the selective *I_h_* antagonist ZD7288 (10 µM; n = 5). Control ZAP-induced voltage traces are compared to those in the presence of ZD7288, at −75 mV (A1) and at −65 mV (A2). The corresponding impedance profiles are shown in A3 (Q = 1.25 at *f_res_* = 2.35 Hz, for control and Q = 1.00 for ZD7288; according to data fit by the theoretical curve, see Methods) and A4 (Q = 1.07 at *f_res_* = 1.8 Hz, for control and Q = 1.00, for ZD7288), respectively. ZAP amplitude was 10 pA for control recordings, and 7/10 pA for ZD7288 at −75/−65 mV. See text for average data from the 5 experiments, indicating that resonance essentially disappears in both potential ranges. *B1*–*B4*, Application of the selective KCNQ channel blocker XE991 (10 µM; n = 7) to a different neuron revealed a contribution of *I_m_* at depolarized potentials. Voltage responses to ZAP stimuli applied at −75 mV (B1) and at −65 mV (B2), before and during the application of XE991. B3 and B4 show the impedance profiles for the traces in B1 and B2, respectively. It can be appreciated an increase in the impedance at low frequencies and the loss of resonance by drug application exclusively in the depolarized voltage range. Q and *f_res_* values in control conditions were, respectively, 1.10 and 2.6 Hz (at −75 mV), and 1.20 and 2.8 Hz (at −65 mV). During XE911 application Q and *f_res_* were 1.10 and 2.6 Hz (−75 mV) and 1.00 and 0.5 Hz (−65 mV). ZAP amplitude was 10 pA for control recordings, and 10/7 pA for ZD7288 at −75/−65 mV. See text for average data from seven experiments.

We first evaluated the effect of the specific *I_h_* blocker ZD7288 on resonant behavior (n = 5). As shown in Figure 5A1 and A3, application of this drug completely abolished resonance at −75 mV. When considering all experiments, the average Q value at this potential was 1.14±0.06 in control conditions, decreasing to 1.01±0.02 in the presence of ZD7288 (p = 0.008; paired t-test, n = 5). This result reproduces the observations made at hyperpolarized potentials during Cs^+^ application and confirms a role of *I_h_* in the generation of subthreshold resonance in ACo neurons. Remarkably, as shown in Figures 4A2 and A4, ZD7288 also removed perithreshold resonance in this neuron. From the 5 neurons studied, only 3 displayed appreciable resonance at −65 mV (Q = 1.14, on average) and in all these cases it was abolished by ZD7288 (Q = 1.00). Therefore, these experiments confirm a contribution of *I_h_* to resonance also at depolarized potentials.

On the other hand, a possible role of *I_m_* in resonance was assessed by bath application of the selective *I_m_* blocker XE991 in 7 resonant neurons. In the example shown in Figures 5B1–B4, XE991 abolished resonance at −65 mV, but not at −75 mV. The average effect of this drug on resonance for the different voltage ranges was as follows: For ZAP stimulation at −75 mV, resonance did not change (Control: 1.14±0.05 and XE991: 1.14±0.08; p = 0.4, paired t-test, n = 7). In contrast, at −65 mV, average Q value decreased from 1.14±0.04 in control conditions, to 1.02±0.03 during XE991 application (p = 0.007, paired t-test, n = 4). However, it was not possible to evaluate resonance at −65 mV in all neurons, since 3 out of 7 cells fired during ZAP stimulation at this potential, but in the remaining 4 experiments, resonance was present in control conditions and it was abolished by XE991 in 3 cases and decreased in the remaining cell. Therefore, on average, XE991 selectively targeted resonance at perithreshold membrane potentials. This treatment had no evident effect on non-resonant cells (n = 7; see Fig. S3B1–B3), confirming a contribution of *I_m_* to the resonant profile at depolarized potentials.

Overall, these results suggest that two different voltage-gated currents participate in subthreshold resonance in ACo neurons: at rest and at membrane potentials hyperpolarized with respect to the resting values, resonant behavior relies on *I_h_*, while at perithreshold potentials, both *I_h_* and *I_m_* can contribute to resonance. The fact that most ACo resonant neurons presented resonance at hyperpolarized potentials, together with our observations with Cs^+^ and ZD7288, point to *I_h_* as a major generator of subthreshold resonance in this region. However, cases as that shown in Figure 5B, where *I_m_* appears also to be key at perithreshold conditions, raises the question of whether the contribution of *I_m_* is also a general property of resonant cells. As the activation voltage of *I_m_* lies close to action potential threshold, it is often difficult to resolve the contribution of this current to subthreshold resonance. Also, our pharmacological experiments indicate that the presence of perithreshold resonance does not necessarily imply that *I_m_* is always contributing, as it could also be generated by *I_h_*. Therefore, we performed experiments in which TTX was applied to the external solution to avoid action potential discharges and in this way reach more depolarized potentials to optimize the conditions to observe *I_m_*-dependent resonance [26]. We found that *I_m_*-resonance was actually not present in all resonant cells; two examples are presented in Figures S4 and S5. As expected, after TTX application resonance was decreased but not abolished at −65 mV (Fig. S4). However, resonance completely disappeared at more depolarized potentials (−50 and −40 mV). In a similar experiment, the application of external Cs^+^ in addition to TTX eliminated *I_h_*-dependent resonance, leaving no signs of resonant behavior from hyperpolarized voltages up to −43 mV (Fig. S5). For comparison, we reproduced results from CA1 pyramidal cells showing that in the presence of TTX, *I_m_*-mediated resonance was observed in every cell [26], and confirmed that resonant behavior at depolarized (−45 mV) but not hyperpolarized (−75 mV) potentials was eliminated by XE991 in these cells (see Fig. S7).

Thus, our experimental results suggest the existence of a variety of resonant neurons in ACo. This diversity may be due to a differential expression of resonance-generating conductances in the cells.

### Computer simulations with a conductance-based model reproduce both sub- and suprathreshold dynamics

A limitation of the phenomenological model (RLC model) is that it only applies to small voltage fluctuations, for which the effects of membrane nonlinearities are not significant [38], [39]. This limitation precludes the use of this approximation in the suprathreshold regime. Moreover, even in subthreshold conditions it is not straightforward to relate the inductor branch parameters with the biophysical properties of the different voltage-gated conductance types involved in resonance. Therefore, as an independent and entirely different approach to examine the contribution of the three mentioned voltage-dependent currents to the subthreshold resonant profiles, we developed a comprehensive conductance-based membrane model using a classical Hodgkin and Huxley formalism (see Methods). In particular, the model was expected to clarify to what extent the presence of *I_h_* and/or *I_m_* in different resonant neurons could account for the diverse resonance patterns that we observed. In addition to *I_h_*, *I_m_* and *I_NaP_*, our single compartment model included a leak current *I_leak_* and the two spike-generating currents *I_Na,H_* and *I_K,H_*, to explore the impact of subthreshold membrane resonance on neuronal spiking. The functions describing the kinetics and voltage dependence of the different currents were taken from previous studies in cortical neurons [22], [41], [44] and are displayed in Tables 1 and 2. Reversal potentials for Na^+^ or K^+^-selective currents were calculated from the ionic concentrations used in the experiments and are also shown in Table 1. We explored the parameters space (maximal conductance values g*_leak_*, *g_h_*, g*_m_* and *g_NaP_*; the voltage dependence and relaxation times were not modified from published results) while keeping the cell capacitance and input resistance (dependent on the leak and hyperpolarization-dependent conductances) in accordance to the ranges observed in the experiments. An additional constraint for parameter setting was the reproduction of the average resting membrane potential. A list of the parameters used in the simulations is presented in Table 3. These parameters are of same order of magnitude as those used in previous models for mammalian forebrain neurons [22], [44].

**Table 3:** Parameter settings used for simulations.

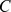 (pF)	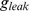 (mS/cm^2^)	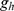 (mS/cm^2^)	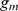 (mS/cm^2^)	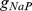 (mS/cm^2^)	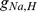 (mS/cm^2^)	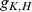 (mS/cm^2^)
50	0.05	0.02	0.06	0.045	17	7.5

**Figure 6:**
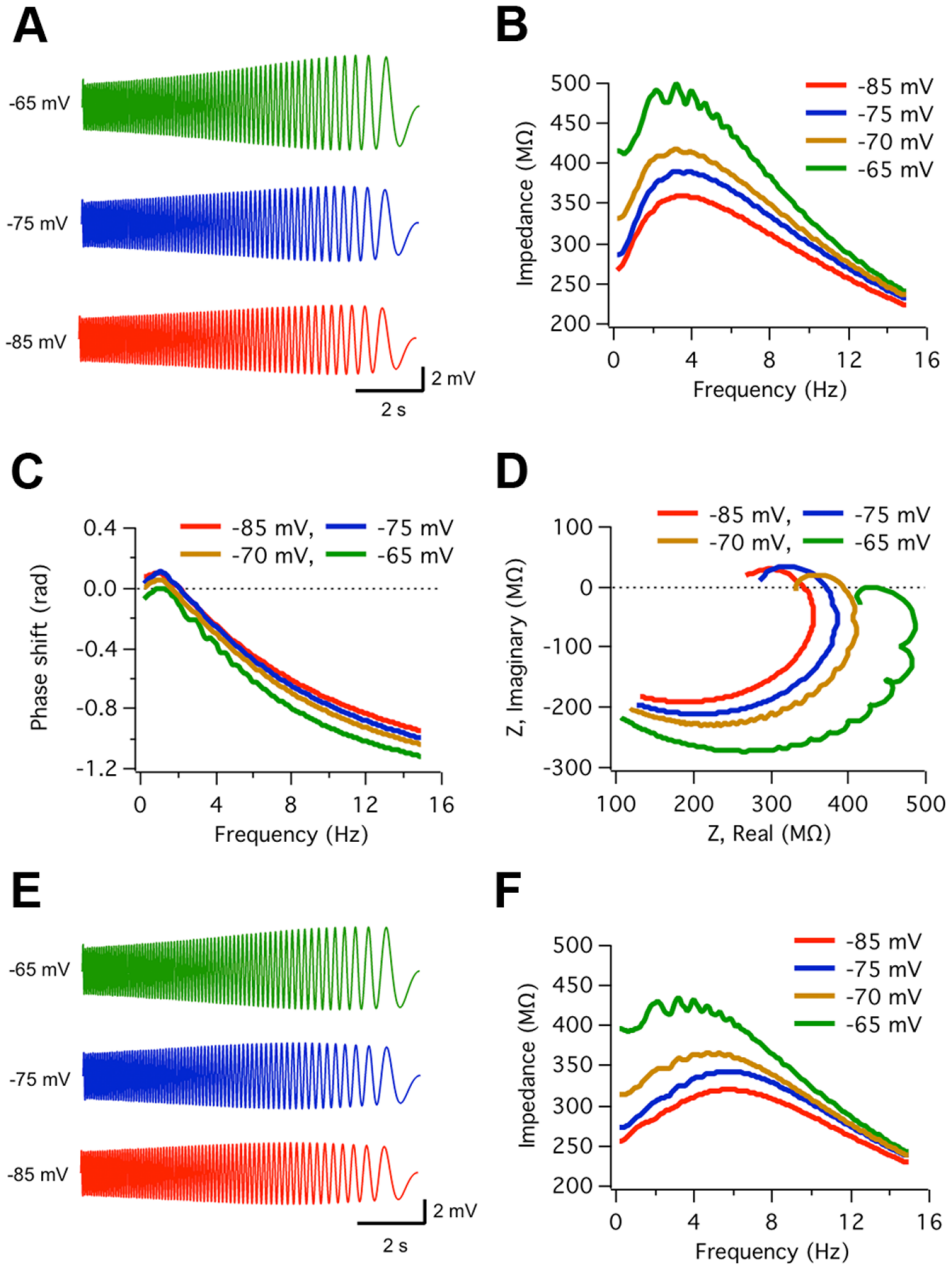
Computer simulations of resonant behavior and the effect of temperature. *A*, Simulated voltage responses to ZAP stimuli (10 pA, 15–0 Hz, at 30°C) applied at three different baseline potentials (NEURON 7.0; see Methods). *B*, Impedance profiles for traces shown in A (and for −70 mV). *f_res_* and Q values are 3.7 Hz and 1.30 (−85 mV), 3.7 Hz and 1.35 (−75 mV), 3.4 Hz and 1.25 (−70 mV), and 3.1 Hz and 1.22 (−65 mV). *C*, Phase shift of the modeled voltage waves relative to ZAP current waves, as a function of frequency. *D*, Complex impedance plot. *E, F*, Same as A, B simulated for 38°C. *f_res_* and Q values are 5.9 Hz and 1.22 (−85 mV), 5.8 Hz and 1.25 (−75 mV), 4.8 Hz and 1.17 (−70 mV), and 3.3 Hz and 1.11, (−65 mV). Model parameters shown in Table 3.

As shown in Figure 6, resonance in ACo neurons was effectively reproduced by the model, as indicated by ZAP-induced voltage waveforms and impedance analysis. In this simulation, we included the two resonant currents *I_h_* and *I_m_*, as our experiments show that both are present in some resonant cells (see Fig. 5B). Figure 6A shows the simulated voltage waves at three different baseline potentials and the corresponding impedance profiles are presented in Figure 6B (the result for −70 mV was additionally included). The phase shift profiles of voltage relative to the injected current are displayed in Figure 6C and the complex impedance representations are shown in Figure 6D. These simulations thus confirmed that together the set of voltage-dependent conductances identified by our pharmacological experiments were able to generate the subthreshold behavior of resonant neurons (see Fig. 1A–D), after imposing the experimentally measured passive parameters of these cells.

As the electrophysiological experiments were conducted at ∼30°C, in most simulations the temperature was set at this value. However, we took advance of the model to explore what would be the frequency selectivity of ACo neurons at the body temperature (∼38°C). Figures 6E and F display the results of these simulations. Interestingly, an increase in *f_res_* was apparent, changing from an average of 3.5 to 5 Hz (see details in the legend of Fig. 6).

**Figure 7:**
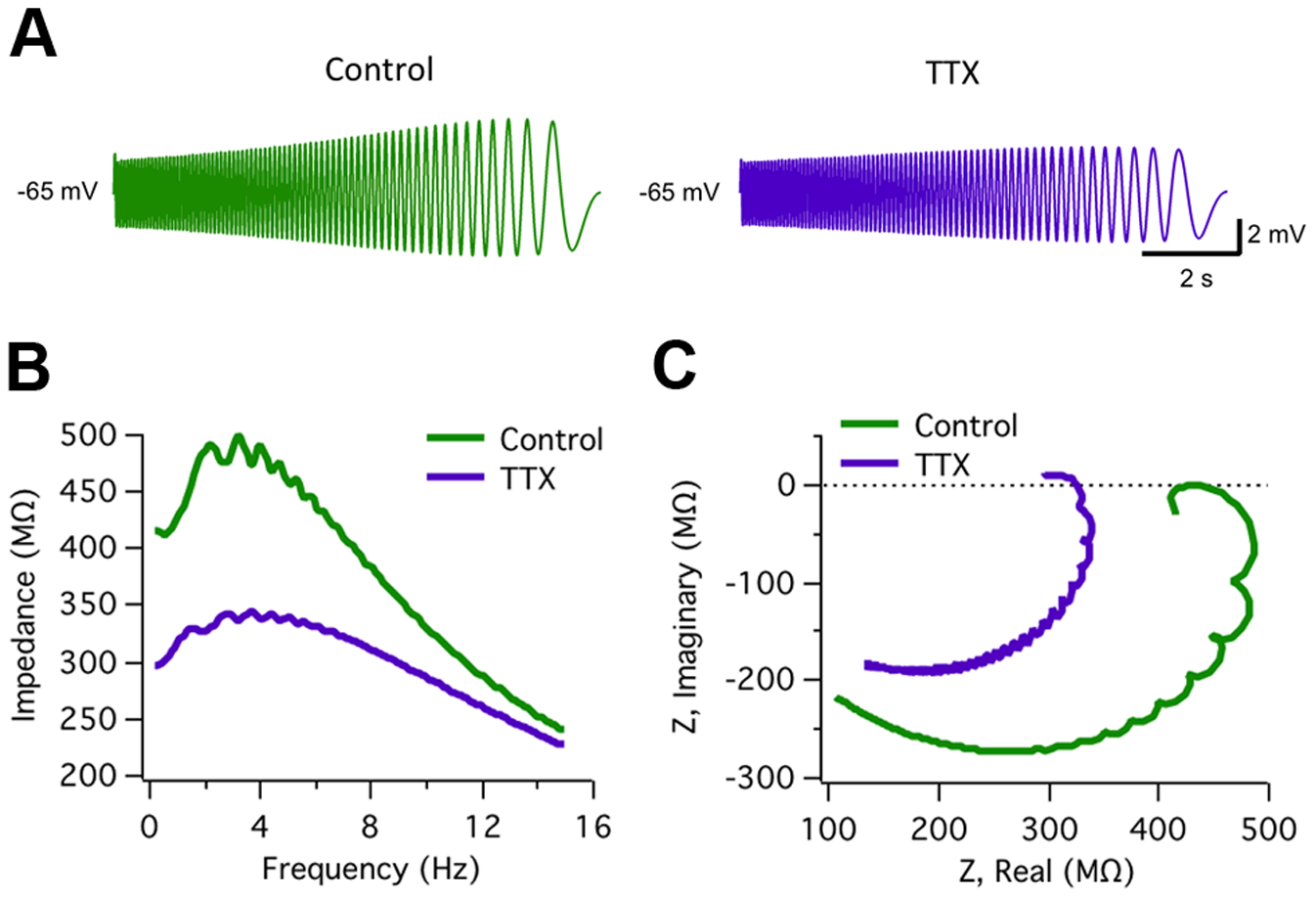
Simulations corroborate amplification role of *I_NaP_*. *A*, Modeled ZAP-generated subthreshold voltage traces in control conditions (parameter settings shown in Table 3) and after mimicking TTX treatment (*g_NaP_*  =  *g_Na,H_*  =  0). *B*, Impedance profiles of the traces in A show an overall reduction in amplitude mainly at lower frequencies and a decreased Q factor (1.12 at 3.8 Hz, in TTX, compared to 1.22 at 3.1 Hz, in control conditions). *C*, Complex impedance plots further demonstrating amplitude reduction but no complete loss of resonant pattern, as indicated by the maintenance of a phase relation with inductive-like properties after TTX application.

With the aim of further dissecting the role of the different conductances in subthreshold responses we used the model to check if the pharmacological results were also reproduced. The effect of each drug was mimicked by setting to zero the maximal conductance value for the target currents. To examine the model prediction for the role of *I_NaP_* in ACo resonance, we explored the effect on voltage waveforms and impedance of abolishing this current. This situation, that mimics TTX application, was simulated by setting the parameter *g_NaP_* (and *g_Na,H_*) to zero. The resulting simulation is shown in Figure 7; a reduction in the amplitude of voltage oscillations is apparent, preferentially at lower frequencies (Fig.7A). As shown in Fig. 7B the impedance resonant pattern was attenuated but not completely abolished. Moreover, at this potential (−65 mV) the phase lag in control conditions reaches zero for low frequencies; interestingly, after removing *I_NaP_* the resonant phase profile not only is preserved but it is even enhanced (Fig. 7C; the mechanism of phase modulation by *I_NaP_* is discussed later). As was the case for the experiments, these results point to an amplifying role of *I_NaP_* in resonance.

**Figure 8:**
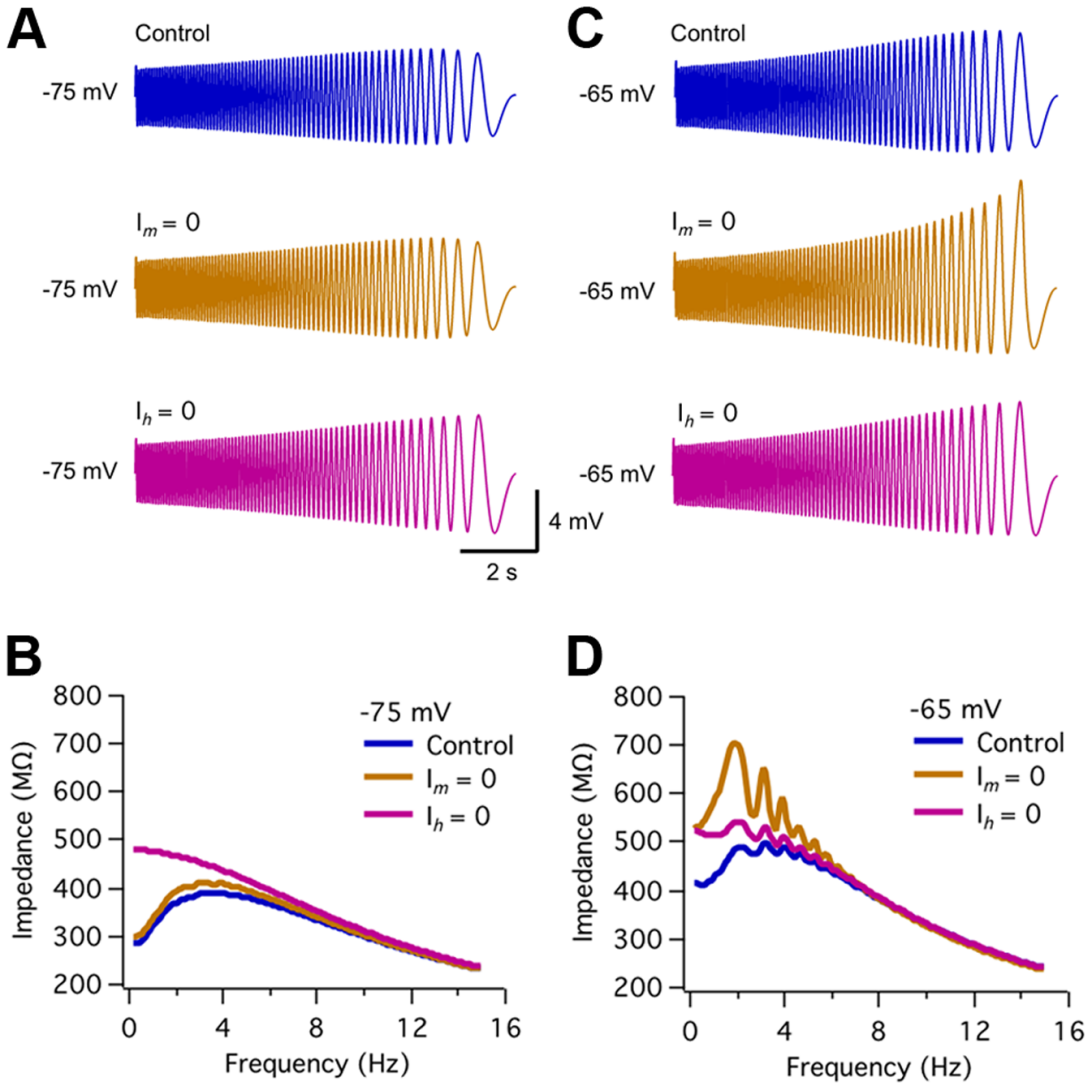
Computer simulations confirm involvement of *I_m_* and *I_h_* in resonance and allow the dissection of their respective contributions at different voltages. *A, B*, Simulated voltage waveforms and impedance profiles generated by ZAP stimulation at −75 mV in control conditions and after elimination of *I_m_* (*g_m_* = 0) or *I_h_* (*g_h_* = 0), respectively. At this voltage resonance was not affected by *I_m_* removal (*f_res_*, Q are 3.7 Hz, 1.35, for control and 3.5 Hz, 1.37, for test conditions). In contrast, *I_h_* elimination completely abolished resonance (Q = 1.00). *C, D*, Same as in A, B, but at −65 mV; *I_m_* removal clearly reduced resonance (Q = 1.12, compared to 1.35 for control; *f_res_* decreased from 3.7 to 2.0 Hz). In turn, resonance was also disrupted after setting *I_h_* = 0 (Q = 1.04; *f_res_* = 1.9 Hz). Parameter settings as in Table 3.

We next examined the differential contribution of *I_h_* and *I_m_* to resonance at various membrane potentials (Fig. 8). At −75 mV the elimination of *I_m_* had no significant effect on voltage response and impedance profile, while removal of *I_h_* abolished resonance (Fig. 8A, B). These results are in agreement with the experimental results shown in Figures 4A1–C1 and 5A1, A3, where selective inhibition of *I_h_* suppressed resonant behavior when the membrane was hyperpolarized. Interestingly, at depolarized potentials (−65 mV) removing *I_h_* abolished resonance (Fig. 8C, D), which is consistent with our experimental results showing a critical contribution of this current to resonance at perithreshold potentials (see Figs. 4B2 and 5A4). On the other hand, setting *I_m_* to zero has an asymmetric effect on the voltage waveform at −65 mV (Fig. 8C, middle trace), as the high-pass filter is completely removed for upward but not for downward voltage incursions. This generates a noisy impedance profile with strongly reduced resonance compared to the control (Fig. 8D; see legend for details). Therefore, this specific simulation indicates that at perithreshold voltages both *I_h_* and *I_m_* can contribute to resonance. The relative contribution of each of these currents among resonant neurons may, however, be variable.

According to our observations ACo resonant neurons are diverse, presenting a variety of resonance intensities, frequencies and voltage-dependence. This may be due to a differential contribution of the ionic currents underlying resonance in these cells. On one extreme, we may consider the existence of neurons -as indicated by our experiments- in which the *I_m_*-dependent mechanism is absent and resonance may rely exclusively on *I_h_* even at depolarized potentials. We explored this possibility by using the model and obtained that in the absence of *I_m_*, resonance can be observed at −65 mV if *g_h_* is increased by a factor of two, i.e. after doubling the density of HCN channels, without additional manipulations of model parameters (see Fig. S7).

In summary, the model successfully replicates subthreshold resonant dynamics and its diversity, and accounts for our pharmacological results as well, thus giving further support to the claim that *I_m_* and *I_h_* differentially contribute to frequency selectivity in ACo neurons, while *I_NaP_* endows the cells with an amplification mechanism that enhances θ resonance.

### Resonant neurons translate subthreshold frequency preference to spiking patterns

**Figure 9:**
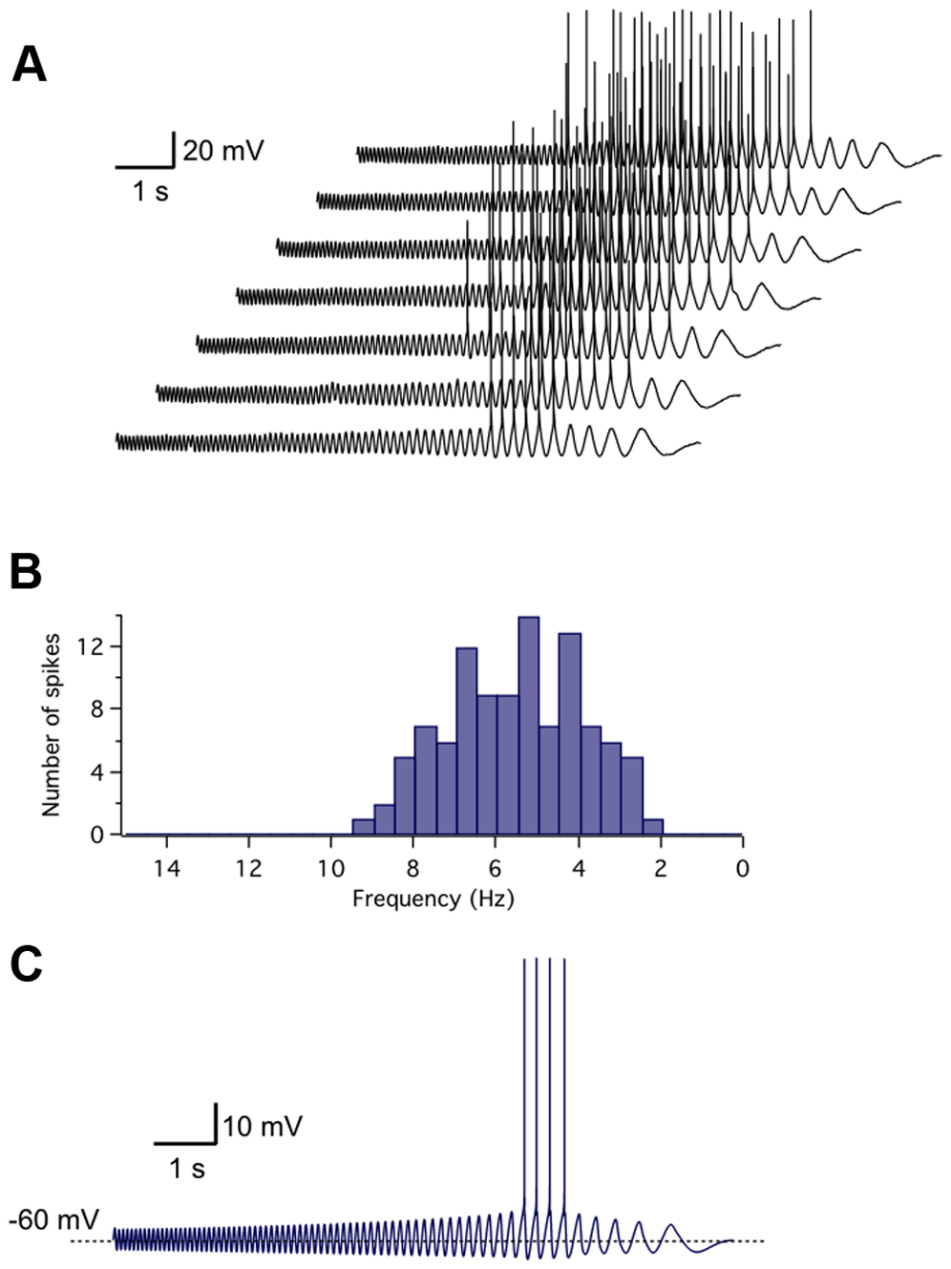
Resonant neurons translate subthreshold frequency preference to spiking regime: experiments and simulations. Representative example from four experiments performed in resonant neurons. *A*, Seven sample responses to the same ZAP stimulus applied to a resonant neuron at a perithreshold voltage, showing the discharge of action potentials for a discrete frequency range of the stimulus. *B*, Histogram of the number of spikes elicited during 13 ZAP stimulations as a function of stimulus frequency, indicating the interval at which discharges were more reliably triggered. *C*, Computer simulation of a resonant neuron response to ZAP stimulation at perithreshold membrane potential. Here the model parameters were set to reproduce the dynamics of this specific neuron, in (mS/cm^2^): g*_leak_* = 0.06, *g_h_* = 0.02, g*_m_* = 0.150, *g_NaP_* = 0.03, where *g_K,H_* = 7.5 and *g_Na,H_* = 17; C = 40 spF.

The different subthreshold voltage responses to oscillatory inputs in resonant and non-resonant neurons suggest that they shall display a completely different behavior during suprathreshold rhythmic stimulation, which may have an impact on processing of incoming inputs. To test this prediction, we examined responses of resonant neurons to ZAP current stimuli applied while cells were basally depolarized to perithreshold levels. Figure 9A shows an example of these experiments (n = 4), in which 7 consecutive perithreshold records from a resonant neuron are displayed. A histogram of the number of spikes elicited as a function of frequency considering all repeats in this neuron (n = 13) is presented in Figure 9B. It can be clearly observed that the resonant cell fires preferentially within a limited interval of frequencies of the oscillatory input and no discharges are elicited at the lowest frequencies. Remarkably, simulations also replicated this selective spiking around the resonance frequency (Fig. 9C), indicating that both subthreshold and suprathreshold resonant dynamics are mimicked by our model.

## Discussion

Here we report the existence of two neuronal populations in layer II of the cortical amygdala, resonant and non-resonant, with clearly different subthreshold membrane potential dynamics. Resonant neurons display an enhanced response to θ-range rhythmic stimuli of a preferred frequency between 2–6 Hz, whereas non-resonant neurons behave as low-pass filters with no frequency preference. Our data indicate that subthreshold frequency preference, or resonance, results from the existence of two active low-frequency filter mechanisms that reduce the response to slow oscillatory stimuli. These mechanisms are independent, one acting in the whole subthreshold voltage range and relying on *I_h_* (that flows through HCN channels) and the other effective only at perithreshold potentials, generated by the muscarine-sensitive K^+^ current *I_m_* (depending upon KV7/KCNQ channels). The resonance is boosted by a persistent Na^+^ current, *I_NaP_*.

### The mechanisms of subthreshold resonance in ACo

In general, resonance requires the coexistence of both high-pass and low-pass filter mechanisms in the cells [15]. The high-pass filter involves slow voltage-dependent currents that are substantially activated only in the low frequency range and that when activated reduce the amplitude of voltage changes, decreasing the membrane impedance. In ACo neurons, the voltage dependence of *I_h_* and *I_m_* and their activation/deactivation time constants determine the frequency preference range. HCN channels slowly activate by membrane hyperpolarization giving rise to a net inward cationic current that promotes cell depolarization towards its reversal potential (−40 mV). In turn, KV7/KCNQ channels are closed at resting potential and are activated by depolarization, originating an outward K^+^ current that hyperpolarizes the cell back to its resting level. Oscillatory stimulation with a shorter period than the activation times of *I_h_* or *I_m_* will activate these currents, with the consequent shunting of the voltage deflections, hence acting as high-pass filters. For faster oscillatory stimuli the time needed to charge the membrane acts as a low-pass filter. The combination of these two filter mechanisms produces a voltage response with a maximal value determined by the tuning effect resulting from the time constant of the resonant currents and their passive membrane properties (resistance and capacitance).

The voltage dependence and kinetics of resonance-generating conductances also have an impact on the phase profile (Fig. 4). The RLC phenomenological model offers a simplified alternative to simulate this effect, but it does not allow a straightforward understanding of what are the biophysical properties responsible for the phase change. As for the reduction in the amplitude of voltage oscillations at low frequencies, the effect on the phase results from the complex and dynamical interplay between the different ionic currents present in resonant cells and the membrane voltage, during the application of an external oscillatory current (see Eq. 3). In this context, the delayed voltage-dependent activation/deactivation of *I_h_* (∼100 ms at 30°C) is critical, as what determines the activation level of *I_h_* at a specific time is the voltage value ∼100 ms earlier. To illustrate how this property affects the phase, we can compare the time at which the ZAP-induced voltage wave crosses the middle line (baseline voltage set by the injected DC current), in the presence or absence of *I_h_*. In the first case, the activation of *I_h_* will be high when approaching the middle line during depolarizing incursions (for frequencies around or below *f_res_*), because the membrane underwent the maximal level of hyperpolarization about a quarter of cycle earlier. Thus, when comparing the middle-line crossing times in the presence or absence of HCN channels, it is expected that these channels will fasten the depolarization process and the voltage will cross this level earlier. In turn, during repolarization *I_h_* will exert a damping effect, but it will be comparatively weak due to deactivation of this current after reaching the voltage peak. Therefore, the presence of *I_h_* leads to a reduction in the voltage wave lag relative to the current wave (impedance phase). Remarkably, depending on the relative contributions of inductive and passive membrane properties, and on the input frequency, the impedance phase may become positive, meaning that the voltage wave precedes the current wave (Fig.1C).

Interestingly, an effect of the short activation delay of *I_NaP_* (∼5 ms) on phase can also be resolved in our experiments. This effect goes in the opposite direction and mainly affects oscillations in the higher frequency range: as demonstrated by the increase in the phase caused by TTX at higher frequencies (Fig. 3C).

To our knowledge, this is the first time that a dual subthreshold resonant mechanism is described in neurons from the amygdaloid complex (even though this duality is not observed in all ACo cells). Previous work characterizing subthreshold behavior of basolateral amygdala neurons in guinea pig found resonance at depolarized membrane potentials, with a peak frequency of 2.4 Hz, that was attributed to an m-type current [49]. In that work resonance was only explored at membrane potentials more positive than −70 mV, for which it is likely that the HCN conductance may have not been sufficiently activated. However, no rectification was detected in the hyperpolarizing direction, suggesting that these channels are not significantly expressed in the cells studied. Conversely, recent work in the rat supports a contribution of *I_h_* to subthreshold resonance in the basolateral amygdala [25]. Although species differences may explain these discrepancies, further work is needed to elucidate whether the dual resonant behavior at θ frequency described here for rat ACo neurons is shared by other regions of the amygdaloid complex.

Subthreshold resonance relying on either *I_h_* or *I_m_* has been reported in different regions of the rodent brain. In the rat subiculum *I_h_*–dependent resonance is observed at resting and hyperpolarized potentials, while no resonant behavior is observed at depolarized voltages [19]. In the EC frequency preference requires *I_h_*, with *I_m_* playing a modulatory role [20], [21]. On the other hand, an *I_m_*–dependent resonance mechanism exists in frontal neurons from guinea pig [22], but not from the rat, where it relies on *I_h_* [23]. A dual subthreshold resonance mechanism similar to that reported here for ACo has only been found in rat hippocampal CA1 pyramidal neurons [26], suggesting a common strategy for neural activity orchestration or signal filtering in these two learning-related brain regions [14], [15], although they have some differences that are discussed below. In both CA1 and ACo neurons θ resonance implicates HCN and KV7/KCNQ channels, responsible for *I_h_* and *I_m_*, respectively [16], [18].

We are also reporting a contribution of *I_NaP_* to resonance amplification in ACo neurons. A similar role plays *I_NaP_* in the rat basolateral amygdala, the EC, the frontal cortex and the hippocampus [9], [19], [22], [23], [26], [50]. We show that non-resonant neurons also possess the *I_NaP_* current, therefore this regenerative voltage-dependent mechanism would contribute to further differentiate the responsiveness of the two populations: while in resonant cells *I_NaP_* would preferentially amplify the largest voltage deflections generated for *f_res_*, in non-resonant cells it would mainly enhance the voltage changes at the lowest frequencies, those that are filtered in the first neuronal subpopulation.

We previously reported that a high fraction of layer II ACo neurons displays intrinsic TTX-sensitive MPOs [8]. While the mechanisms underlying MPOs and resonance are thought to be related, several observations suggest they are not identical [15]. In contrast to the effect on resonance, TTX completely abolished oscillations in ACo but only attenuated resonant behavior; similar observations have been made in neocortical neurons [22]. This suggests that *I_NaP_* plays a critical role in the generation of MPOs, while playing mainly an amplifying role in resonance. Accordingly, MPOs are absent at resting and hyperpolarized membrane potentials, but this is not the case for resonance. Finally, while in ACo the frequency of MPOs strongly increases with depolarization [8], resonance frequency is comparatively poorly voltage-dependent (see Fig. 2 for average values). Here we did not systematically compared the incidence of MPOs and resonance in the same neurons, however, the differences in the percentage of ACo resonant neurons (54%) and those displaying oscillations (68%) is not surprising, as both phenomena are not necessarily expressed in the same cells [15].

The frequency preference range of ACo neurons (Fig. 2) and CA1 pyramidal neurons [26] is comparable in experiments conducted at a similar temperature. While we did not examine the temperature dependence of *f_res_*, our model predicts a frequency shift of about 2 Hz by an increase in temperature from 30 to 38°C. This is in agreement with experimental results from the hippocampus [34], [43] and matches the frequency of *in vivo* θ waves [51]. Nevertheless, differences arise when comparing resonance in ACo and CA1. Firstly, while in CA1 virtually all pyramidal neurons display θ frequency selectivity, we distinguish two main ACo cell populations, resonant and non-resonant. As our criterion to discriminate among these cell types was more stringent (Q≥1.10 compared to Q>1.00 used in [26]), it is possible that the number of ACo resonant neurons was underestimated. However, the existence of an important population of cells lacking low-frequency filtering properties results apparent after examining their impedance profiles (Q = 1.00; Fig. 2B) and their insensitivity to Cs^+^ and XE991. A second relevant difference is that while resonance in CA1 neurons virtually disappears at resting membrane potential, ACo neurons exhibit resonance in the whole subthreshold voltage range. This is probably due to the fact that, in contrast to CA1 cells, the contribution of *I_h_* to resonance in ACo neurons is significant in the whole subthreshold range. The question whether this may be related to differences in the voltage-dependence of HCN channels [52] remains to be investigated. Finally, the dual mechanism was found in all CA1 pyramidal cells [26], but our evidence indicates that a subpopulation of resonant ACo cells lack the *I_m_*–dependent mechanism.

Overall, our results suggest that, in contrast to other brain regions in which θ resonance has been studied, ACo neurons exhibit different contributions of the two resonance-generating mechanisms, including cells expressing either both, only one or none of them.

### Functional implications of θ-frequency resonance

Whether the intrinsic frequency preference of mammalian resonant neurons plays a role in the generation or spreading of orchestrated rhythmic activity in the brain is still an open question. A condition that has made the resolution of this subject elusive is that resonant neurons are mainly present in high-order processing regions, such as the hippocampus, the neocortex and the basolateral amygdala, where they receive extremely complex signal patterns influenced by different sensory modalities. Therefore, it is difficult to recreate in experimental conditions the activity patterns that these neurons receive *in vivo* and to evaluate the transfer function and filter properties that they display in the brain. This context makes the cortical amygdala a promising model to evaluate a possible functional role for θ frequency resonance, because as the ACo receives direct projections from the OB, it is expected that the afferent activity preserves the characteristic sniffing-driven rhythmicity of the olfactory circuit [28]. The impact of the intrinsic frequency preference on neuronal and network dynamics upon such oscillatory drive remains to be determined. Resonant neurons may impose their individual frequency preference to the processed signals, excluding (filtering) other rhythmic inputs and selectively transmitting activity at their individual resonant frequency, thus working as frequency discriminators (resonators) [53]. This may constitute a mechanism for selective communication between neurons. Intrinsic resonance in the EC has been related to the existence of a cognitive spatial map represented in a sequence of grid-like patterns, in which grid spacing correlates with the *f_res_* of stellate neurons which is scaled along the dorso–ventral axis [54]. Finally, resonance may contribute to generate or propagate network rhythms [14], [15].

Our observation that cortical amygdala neurons translate their subthreshold frequency preference to a spiking regime indicates that the spike-triggering machinery is coupled to the subthreshold resonance mechanism (the voltage for spike-generating Na^+^ channel activation partially overlaps with the voltages at which *I_m_*/*I_s_* filter slow oscillations). Therefore, resonant ACo neurons are endowed with intrinsic electrophysiological properties that make them able to selectively generate and propagate rhythmic activity at their resonance frequency.

In contrast to CA1 pyramidal neurons, the dual mechanism of resonance is not general in ACo resonant neurons. Indeed, our study shows the existence of an inhomogeneous population of resonant cells, suggesting a complex signal processing in this olfactory region. Previous anatomical studies proposed that this region constitutes an intermediate formation among cortical and nuclear structures [55]. It can be speculated that different populations of resonant neurons in ACo may be activated depending on their specific afferent connectivity or on the sniffing pattern. Further studies are needed to explore these possibilities. Moreover, it should be noted that the layer II cells recorded here may include both projection neurons and interneurons, thus additional work is required to evaluate if different mechanisms of resonance (or the absence of this property) are associated to specific neuronal types in ACo.

While neuronal resonance is usually studied upon somatic injection of oscillatory currents, the real effect of rhythmic synaptic drive on cellular activity also depends on synaptic and dendritic filter features. Synaptic filtering depends on the specific short-term plasticity properties (mainly presynaptic) of the studied connections [53], [56]. On the other hand, distal dendritic resonance during local injection of oscillatory currents has been demonstrated in neocortical and hippocampal pyramidal cells; the dendro-somatic transfer impedance curve preserves the resonant profile, indicating that dendritic filtering does not eliminate frequency selectivity [57], [58]. The cellular distribution of resonance-related conductances also shapes the effect of resonance on neuronal activity. In CA1 pyramidal neurons, the differences in localization and voltage activation range of these channels endow the cells with two segregated and independent cellular compartments for frequency preference and processing of incoming signals: dendrites resonate at hyperpolarized potentials (through HCN channels), whereas somatic filtering occurs at depolarized potentials (KV7/KCNQ channels) [59], [60].

Finally, some authors have found that recreation of the *in vivo* conditions in brain slices by mimicking synaptic bombardment through a reduction in membrane resistance, dampens the intrinsic frequency preference of resonant neurons [61]. While this possibility shall be examined in the cortical amygdala, the comparatively high basal input resistance of ACo resonant cells suggests that these neurons may preserve their filter properties and translate the subthreshold frequency preference into spikes when resistance drops *in vivo* due to synaptic activity (by ∼50% [62]).

### Olfactory coding, motivation and learning

Anatomical data suggest that the olfactory representations in the PC and olfactory amygdala are differentially organized. The spatial map characterizing the OB is lost in this cortex because the projections from one glomerulus to the PC are diffuse. In contrast, the projections from the different glomeruli to the cortical amygdala are organized into spatially stereotyped overlapping patches [63]. The cortical amygdala receives inputs from the entire OB, but in contrast to PC, there is a bias to dorsal glomeruli [64], shown to convey inputs required for innate responses to aversive odorants [65]. These observations and the fact that olfactory tract stimulation induces a wave of activity in PC and EC that converges in the olfactory amygdala [66], suggest that this structure plays a distinct, mostly unexplored role in olfactory integration.

Behavioral and electrophysiological studies in the olfactory system indicate that a single sniff is sufficient for fine odor discrimination [67]. However, there are differences in the encoding strategies for odor identification depending on whether or not it has a behavioral value (for example, is it rewarded or not?). In the rodent OB and PC, odor identity is respectively encoded by the latency and the rate of the evoked activity phase-locked to the first sniff [28], [68]. In contrast, if the odor has a behavioral value, in both olfactory regions odor information is conveyed by the non-phase-locked firing rate during several sniffing cycles [29], [69]. It should be noted that it is the increased synchronized activity of groups of neurons what is critical for encoding odor value during an active detection task, however, during passive detection odor identity is strongly conveyed by spiking activity phase-looked to sniffing [69]. In a similar way, the formation and expression of auditory emotional memories involve an increase in synchronized firing and rhythmic activity at the θ-resonance frequency of neurons in the lateral amygdala [70], [71]. Therefore, when the identification of an odor has biological relevance, additional high-order structures involved in emotional memory formation/retrieval may be recruited; our results point to the cortical amygdala as an attractive candidate endowed with θ rhythmic properties. In this context, the role of the cortical amygdala in olfactory integration may be related to innate or learned odor preference or aversion, based on a selective filtering of oscillatory activity.

## Supporting Information

Figure S1 **Q value and peak frequency distributions for different voltage ranges.** *A–D*, Histograms of the Q factors (left) and frequencies at which Z reaches its maximum (peak frequency; right), for ZAP stimulation at different membrane potentials binned every 10 mV (averages: −85, −75, −65 and −55 mV). Data with Q<1.10 are shown in black and those with Q≥1.10, in red. The number of recorded cells per voltage range was 125, 132, 92 and 41, for −85, −75, −65 and −55 mV, respectively.
(TIF)

Figure S2 ***I_NaP_*** **is also present in non-resonant cells.** Effect of TTX (1 µM) on the subthreshold responses of a non-resonant neuron. *A1*, ZAP-stimulation-evoked voltage responses before (Control) and during the extracellular application of TTX (A2). *B*, Impedance profiles, before and during TTX treatment.
(TIF)

Figure S3 **Cs^+^ and XE991 do not alter impedance profiles of non-resonant neurons.** *A*, ZAP-induced voltage traces at a baseline potential of −80 mV from a non-resonant neuron before (A1) and during the bath application of 4 mM Cs^+^ (A2). A3, Normalized impedance profiles for the recordings in A1 and A2. *B*, ZAP-induced voltage traces from a non-resonant neuron before (B1) and during application of 10 µM XE991 (B2) at a baseline membrane potential of −61 mV. *B3*, Impedance profiles for recordings in B1 and B2.
(TIF)

Figure S4 **Spike blockade in a neuron resonating at subthreshold voltages revealed the absence of** ***I_m_*** **-dependent resonance at more depolarized potentials.** *A*, ZAP-induced voltage traces at control conditions and after application of TTX to allow exploration of resonance at suprathreshold potentials. Resonance is observed at −65 mV in both conditions, but it is absent at voltages at which *I_m_* is expected to be active and *I_h_* non active (−50 or −40 mV, compare with Figure S6). *B*, Impedance profiles for traces in A (Q = 1.25 for control and 1.08 in TTX at −65 mV; Q = 1.00 for more depolarized voltages).
(TIF)

Figure S5 **Blockade of** ***I_h_*** **-resonance and spikes confirms the lack of the** ***I_m_*** **-dependent mechanism in another ACo neuron.** *A*, ZAP-induced voltage traces at −89 and −65 mV in control conditions (Q and *f_res_* are 1.2 at 3.7 Hz and 1.4 at 2.9 Hz, respectively). *B*, To evaluate the existence of *I_m_*-dependent resonance in this neuron, voltage traces were recorded in the presence of TTX (to block spikes) and Cs^+^ (4 mM; to eliminate *I_h_*-dependent resonance). Subthreshold resonance was completely eliminated and it is absent even at −43 mV where *I_m_* is supposed to be fully active (compare with Figure S6). C and D, impedance profiles for traces in A and B, respectively.
(TIF)

Figure S6 **Example of a hippocampal resonant neuron showing a strong** ***I_m_*** **-dependent resonance at suprathreshold potentials in TTX.** *A*, ZAP-induced voltage traces under TTX and after the application of 10 µM XE991 to block KCNQ channels, at −75 mV (A1) and at −45 mV (A2). *B*, impedance profiles for the recordings in (A) showing that resonance at this hyperpolarized potential is not affected by XE991 (B1). In contrast, at the suprathreshold potential (B2) application of the KCNQ blocker confirmed that resonance relies completely on *I_m_* at this voltage.
(TIF)

Figure S7 **Computer simulations confirm that perithreshold resonance can be generated exclusively by** ***I_h_*** **.** *A*, Simulated voltage responses to ZAP stimuli (10 pA, 15-0 Hz, at 30°C) applied at −85, −75 and −65 mV baseline potentials (NEURON 7.0; see Methods). B, Impedance profiles for the traces in A (including also the result for −70 mV). *f_res_* and Q values are 4.6 Hz and 1.58 (−85 mV), 4.5 Hz and 1.73 (−75 mV), 3.8 Hz and 1.57 (−70 mV), and 2.8 Hz and 1.4 (−65 mV). Model parameters as shown in Table 3, but with *g_h_* = 0.04 mS/cm^2^ and *g_m_* = 0).
(TIF)

## References

[1] McDonald AJ (1998) Cortical pathways to the mammalian amygdala. Prog Neurobiol 55: 257–332. 9643556 10.1016/s0301-0082(98)00003-3

[2] Swanson L, Petrovich L (1998) What is the amygdala? Trends Neurosci 21: 323–331. 9720596 10.1016/s0166-2236(98)01265-x

[3] Pitkänen A, Savander V, LeDoux JE (1997) Organization of intra-amygdaloid circuitries in the rat: an emerging framework for understanding functions of the amygdala. Trends Neurosci 20: 517–523. 9364666 10.1016/s0166-2236(97)01125-9

[4] Ledoux JE (2000) Emotion Circuits in the Brain. Annu Rev Neurosci 23: 155–184. 10845062 10.1146/annurev.neuro.23.1.155

[5] Sah P, Faber ESL, Lopez De Armentia M, Power J (2003) The amygdaloid complex: anatomy and physiology. Physiol Rev 83: 803–834. 12843409 10.1152/physrev.00002.2003

[6] Paxinos G, Kus L, Ashwell K, Watson C (1999) Chemoarchitectonic atlas of the rat forebrain. Academic Press.

[7] Sevelinges Y, Gervais R, Messaoudi B, Granjon L, Mouly AM (2004) Olfactory fear conditioning induces field potential potentiation in rat olfactory cortex and amygdala. Learn Mem 11: 761–769. 15537739 10.1101/lm.83604 PMC534705

[8] Sanhueza M, Bacigalupo J (2005) Intrinsic subthreshold oscillations of the membrane potential in pyramidal neurons of the olfactory amygdala. Eur J Neurosci 22: 1618–1626. 16197502 10.1111/j.1460-9568.2005.04341.x

[9] Pape HC, Paré D, Driesang RB (1998) Two types of intrinsic oscillations in neurons of the lateral and basolateral nuclei of the amygdala. J Neurophysiol 79: 205–216. 9425192 10.1152/jn.1998.79.1.205

[10] Leung LW, Yim CY (1991) Intrinsic membrane potential oscillations in hippocampal neurons in vitro. Brain Res 553: 261–274. 1718544 10.1016/0006-8993(91)90834-i

[11] Alonso A, Llinás R (1989) Subthreshold Na-dependent theta-like rhythmicity in stellate cells of entorhinal cortex layer II. Nature 342: 175–177. 2812013 10.1038/342175a0

[12] Leung LS, Yu H (1998) Theta-frequency resonance in hippocampal CA1 neurons *in vitro* demonstrated by sinusoidal current injection. J Neurophysiol 79: 1592–1596. 9497437 10.1152/jn.1998.79.3.1592

[13] Haas JS, White JA (2002) Frequency selectivity of layer II stellate cells in the medial entorhinal cortex. J Neurophysiol 88: 2422–2429. 12424283 10.1152/jn.00598.2002

[14] Llinás R (1988) The intrinsic electrophysiological properties of mammalian neurons: insights into central nervous system function. Science 242: 1654–1664. 3059497 10.1126/science.3059497

[15] Hutcheon B, Yarom Y (2000) Resonance, oscillation and the intrinsic frequency preferences of neurons. Trends Neurosci 23: 216–222. 10782127 10.1016/s0166-2236(00)01547-2

[16] Biel M, Wahl-schott C, Michalakis S, Zong X (2009) Hyperpolarization-activated cation channels: From genes to function. Physiol Rev 89: 847–885. 19584315 10.1152/physrev.00029.2008

[17] Brown D (1988) M-currents: an update. Trends Neurosci 11: 294–299. 2465631 10.1016/0166-2236(88)90089-6

[18] Wang HS, Pan Z, Shi W, Brown BS, Wymore RS, et al (1998) KCNQ2 and KCNQ3 potassium channel subunits: molecular correlates of the M-channel. Science 282: 1890–1893. 9836639 10.1126/science.282.5395.1890

[19] Wang W-T, Wan Y-H, Zhu J-L, Lei G-S, Wang Y-Y, et al (2006) Theta-frequency membrane resonance and its ionic mechanisms in rat subicular pyramidal neurons. Neuroscience 140: 45–55. 16527421 10.1016/j.neuroscience.2006.01.033

[20] Boehlen A, Henneberger C, Heinemann U, Erchova I (2013) Contribution of near-threshold currents to intrinsic oscillatory activity in rat medial entorhinal cortex layer II stellate cells. J Neurophysiol 109: 445–463. 23076110 10.1152/jn.00743.2011 PMC3545459

[21] Nolan MF, Dudman JT, Dodson PD, Santoro B (2007) HCN1 channels control resting and active integrative properties of stellate cells from layer II of the entorhinal cortex. J Neurosci 27: 12440–12451. 18003822 10.1523/JNEUROSCI.2358-07.2007 PMC6673323

[22] Gutfreund Y, Yarom Y, Segev I (1995) Subthreshold oscillations and resonant frequency in guinea-pig cortical neurons: physiology and modelling. J Physiol 483 3: 621–640. 7776248 10.1113/jphysiol.1995.sp020611 PMC1157807

[23] Hutcheon B, Miura RM, Puil E (1996) Subthreshold membrane resonance in neocortical neurons. J Neurophysiol 76: 683–697. 8871191 10.1152/jn.1996.76.2.683

[24] Pape H, Driesang RB (1998) Ionic mechanisms of intrinsic oscillations in neurons of the basolateral amygdaloid complex. J Neurophysiol 79: 217–226. 9425193 10.1152/jn.1998.79.1.217

[25] Ehrlich DE, Ryan SJ, Rainnie DG (2012) Postnatal development of electrophysiological properties of principal neurons in the rat basolateral amygdala. J Physiol 590: 4819–4838. 22848043 10.1113/jphysiol.2012.237453 PMC3487039

[26] Hu H, Vervaeke K, Storm JF (2002) Two forms of electrical resonance at theta frequencies, generated by M-current, h-current and persistent Na^+^ current in rat hippocampal pyramidal cells. J Physiol 545: 783–805. 12482886 10.1113/jphysiol.2002.029249 PMC2290731

[27] Huerta PT, Lisman JE (1995) Bidirectional synaptic plasticity induced by a single burst during cholinergic theta oscillation in CA1 *in vitro* . Neuron 15: 1053–1063. 7576649 10.1016/0896-6273(95)90094-2

[28] Kepecs A, Uchida N, Mainen ZF (2006) The sniff as a unit of olfactory processing. Chem Senses 31: 167–179. 16339265 10.1093/chemse/bjj016

[29] Doucette W, Gire DH, Whitesell J, Carmean V, Lucero MT, Restrepo D (2011) Associative cortex features in the first olfactory brain relay station. Neuron 69: 1176–1187. 21435561 10.1016/j.neuron.2011.02.024 PMC3064824

[30] Kepecs A, Uchida N, Mainen ZF (2007) Rapid and precise control of sniffing during olfactory discrimination in rats. J Neurophysiol 98: 205–213. 17460109 10.1152/jn.00071.2007

[31] Wesson DW, Donahou TN, Johnson MO, Wachowiak M (2008) Sniffing behavior of mice during performance in odor-guided tasks. Chem Senses 33: 581–596. 18534995 10.1093/chemse/bjn029 PMC2533419

[32] Macrides F, Chorover SL (1972) Olfactory bulb units: activity correlated with inhalation cycles and odor quality. Science 175: 84–87. 5008584 10.1126/science.175.4017.84

[33] Cang J, Isaacson JS (2003) In vivo whole-cell recording of odor-evoked synaptic transmission in the rat olfactory bulb. J Neurosci 23: 4108–4116. 12764098 10.1523/JNEUROSCI.23-10-04108.2003 PMC6741073

[34] Wilson D (2001) Receptive fields in the rat piriform cortex. Chem Senses 26: 577–584. 11418503 10.1093/chemse/26.5.577

[35] Macrides F, Eichenbaum H, Forbes W (1982) Temporal relationship between sniffing and the limbic rhythm during odor discrimination reversal learning. J Neurosci 2: 1705–1717. 7143047 10.1523/JNEUROSCI.02-12-01705.1982 PMC6564372

[36] Puil E, Gimbarzevsky B, Miura RM (1987) Voltage dependence of membrane properties of trigeminal root ganglion neurons. J Neurophysiol 58: 66–86. 3497235 10.1152/jn.1987.58.1.66

[37] Neher E (1992) Correction for liquid junction potentials in patch clamp experiments. Methods Enzymol 207: 123–131. 1528115 10.1016/0076-6879(92)07008-c

[38] Erchova I, Kreck G, Heinemann U, Herz a VM (2004) Dynamics of rat entorhinal cortex layer II and III cells: characteristics of membrane potential resonance at rest predict oscillation properties near threshold. J Physiol 560: 89–110. 15272028 10.1113/jphysiol.2004.069930 PMC1665205

[39] Koch C (1984) Cable theory in neurons with active, linearized membranes. Biol Cybern 50: 15–33. 6324889 10.1007/BF00317936

[40] Hodgkin AL, Huxley AF (1952) A quantitative description of membrane current and its application to conduction and excitation in nerve. J Physiol 117: 500–544. 12991237 10.1113/jphysiol.1952.sp004764 PMC1392413

[41] Spain WJ, Schwindt PC, Crill WE (1987) Anomalous rectification in neurons from cat sensorimotor cortex in vitro. J Neurophysiol 57: 1555–1576. 3585479 10.1152/jn.1987.57.5.1555

[42] French CR, Sah P, Buckett KJ, Gage PW (1990) A voltage-dependent persistent sodium current in mammalian hippocampal neurons. J Gen Physiol 95: 1139–1157. 2374000 10.1085/jgp.95.6.1139 PMC2216358

[43] Adams PR, Brown DA, Constanti A (1982) M-currents and other potassium currents in bullfrog sympathetic neurones. J Physiol 330: 537–572. 6294290 10.1113/jphysiol.1982.sp014357 PMC1225314

[44] Richardson MJE, Brunel N, Hakim V (2003) From subthreshold to firing-rate resonance. J Neurophysiol 89: 2538–2554. 12611957 10.1152/jn.00955.2002

[45] Magee JC (1998) Dendritic hyperpolarization-activated currents modify the integrative properties of hippocampal CA1 pyramidal neurons. J Neurosci 18: 7613–7624. 9742133 10.1523/JNEUROSCI.18-19-07613.1998 PMC6793032

[46] Carnevale NT, Hines ML (2006) The neuron book. Cambridge University Press.

[47] Halliwell JV, Adams PR (1982) Voltage-clamp analysis of muscarinic excitation in hippocampal neurons. Brain Res 250: 71–92. 6128061 10.1016/0006-8993(82)90954-4

[48] Klink R, Alonso A (1993) Ionic mechanisms for the subthreshold oscillations and differential electroresponsiveness of medial entorhinal cortex layer II neurons. J Neurophysiol 70: 144–157. 7689647 10.1152/jn.1993.70.1.144

[49] Pape H, Driesang RB, Popescu AT, Paré D, Rainnie G (1998) Ionic Mechanisms of Intrinsic Oscillations in Neurons of the Basolateral Amygdaloid Complex. J Neurophysiol 79: 217–226. 9425193 10.1152/jn.1998.79.1.217

[50] Burton BG, Economo MN, Lee GJ, White JA (2008) Development of theta rhythmicity in entorhinal stellate cells of the juvenile rat. J Neurophysiol 100: 3144–3157. 18829850 10.1152/jn.90424.2008 PMC2604849

[51] Buzsaki G (2002) Theta oscillations in the hippocampus. Neuron 33: 325–340. 11832222 10.1016/s0896-6273(02)00586-x

[52] Lüthi A, McCormick DA (1999) Modulation of a pacemaker current through Ca(2+)-induced stimulation of cAMP production. Nat Neurosci 2: 634–641. 10404196 10.1038/10189

[53] Izhikevich E (2003) Bursts as a unit of neural information: selective communication via resonance. Trends Neurosci 26: 161–167. 12591219 10.1016/S0166-2236(03)00034-1

[54] Moser EI, Kropff E, Moser MB (2008) Place cells, grid cells, and the brain's spatial representation system. Annu Rev Neurosci 31: 69–89. 18284371 10.1146/annurev.neuro.31.061307.090723

[55] Kalimullina LB, Akhmadeev AV, Minibaeva ZR, Mutalova LR (2004) Structural organization of the amygdaloid complex of the rat brain. Neurosci Behav Physiol 34: 551–555. 15368899 10.1023/b:neab.0000028283.55130.69

[56] Wang X (2010) Neurophysiological and computational principles of cortical rhythms in cognition. Physiol Rev 90: 1195–1268. 20664082 10.1152/physrev.00035.2008 PMC2923921

[57] Narayanan R, Johnston D (2007) Long-term potentiation in rat hippocampal neurons is accompanied by spatially widespread changes in intrinsic oscillatory dynamics and excitability. Neuron 56: 1061–1075. 18093527 10.1016/j.neuron.2007.10.033 PMC2430016

[58] Ulrich D (2002) Dendritic resonance in rat neocortical pyramidal cells. J Neurophysiol 87: 2753–2759. 12037177 10.1152/jn.2002.87.6.2753

[59] Hu H, Vervaeke K, Storm JF (2007) M-channels (Kv7/KCNQ channels) that regulate synaptic integration, excitability, and spike pattern of CA1 pyramidal cells are located in the perisomatic region. J Neurosci 27: 1853–1867. 17314282 10.1523/JNEUROSCI.4463-06.2007 PMC6673553

[60] Hu H, Vervaeke K, Graham LJ, Storm JF (2009) Complementary theta resonance filtering by two spatially segregated mechanisms in CA1 hippocampal pyramidal neurons. J Neurosci 29: 14472–14483. 19923281 10.1523/JNEUROSCI.0187-09.2009 PMC6665813

[61] Fernandez FR, White JA (2008) Oscillations and Periodic Firing in Stellate Cells of the Entorhinal Cortex. J Neurosci 28: 3790–3803. 18385337 10.1523/JNEUROSCI.5658-07.2008 PMC6671103

[62] Destexhe A, Rudolph M, Pare D (2003) The high-conductance state of neocortical neurons in vivo. Nat Rev Neurosci 4: 739–751. 12951566 10.1038/nrn1198

[63] Sosulski DL, Bloom ML, Cutforth T, Axel R, Datta SR (2011) Distinct representations of olfactory information in different cortical centres. Nature 472: 213–216. 21451525 10.1038/nature09868 PMC3354569

[64] Miyamichi K, Amat F, Moussavi F, Wang C, Wickersham I, et al (2011) Cortical representations of olfactory input by trans-synaptic tracing. Nature 472: 191–196. 21179085 10.1038/nature09714 PMC3073090

[65] Kobayakawa K, Kobayakawa R, Matsumoto H, Oka Y, Imai T, et al (2007) Innate versus learned odour processing in the mouse olfactory bulb. Nature 450: 503–508. 17989651 10.1038/nature06281

[66] Kajiwara R, Tominaga T, Takashima I (2007) Olfactory information converges in the amygdaloid cortex via the piriform and entorhinal cortices: observations in the guinea pig isolated whole-brain preparation. Eur J Neurosci 25: 3648–3658. 17610584 10.1111/j.1460-9568.2007.05610.x

[67] Uchida N, Mainen ZF (2003) Speed and accuracy of olfactory discrimination in the rat. Nat Neurosci 6: 1224–1229. 14566341 10.1038/nn1142

[68] Miura K, Mainen ZF, Uchida N (2012) Odor representations in olfactory cortex: distributed rate coding and decorrelated population activity. Neuron 74: 1087–1098. 22726838 10.1016/j.neuron.2012.04.021 PMC3383608

[69] Gire DH, Whitesell JD, Doucette W, Restrepo D (2013) Information for decision-making and stimulus identification is multiplexed in sensory cortex. Nat Neurosci 16: 991–993. 23792942 10.1038/nn.3432 PMC3725200

[70] Paré D, Collins DR (2000) Neuronal correlates of fear in the lateral amygdala: multiple extracellular recordings in conscious cats. J Neurosci 20: 2701–2710. 10729351 10.1523/JNEUROSCI.20-07-02701.2000 PMC6772231

[71] Popa D, Duvarci S, Popescu AT, Léna C, Paré D (2010) Coherent amygdalocortical theta promotes fear memory consolidation during paradoxical sleep. Proc Natl Acad Sci U S A 107: 6516–6519. 20332204 10.1073/pnas.0913016107 PMC2851973

