# Supplementary figures and images for: Electrical Resonance in the θ Frequency Range in Olfactory Amygdala Neurons
Source: PLoS One. 2014 Jan 21;9(1):e85826. doi: 10.1371/journal.pone.0085826 (PMC3897534; doi:10.1371/journal.pone.0085826)

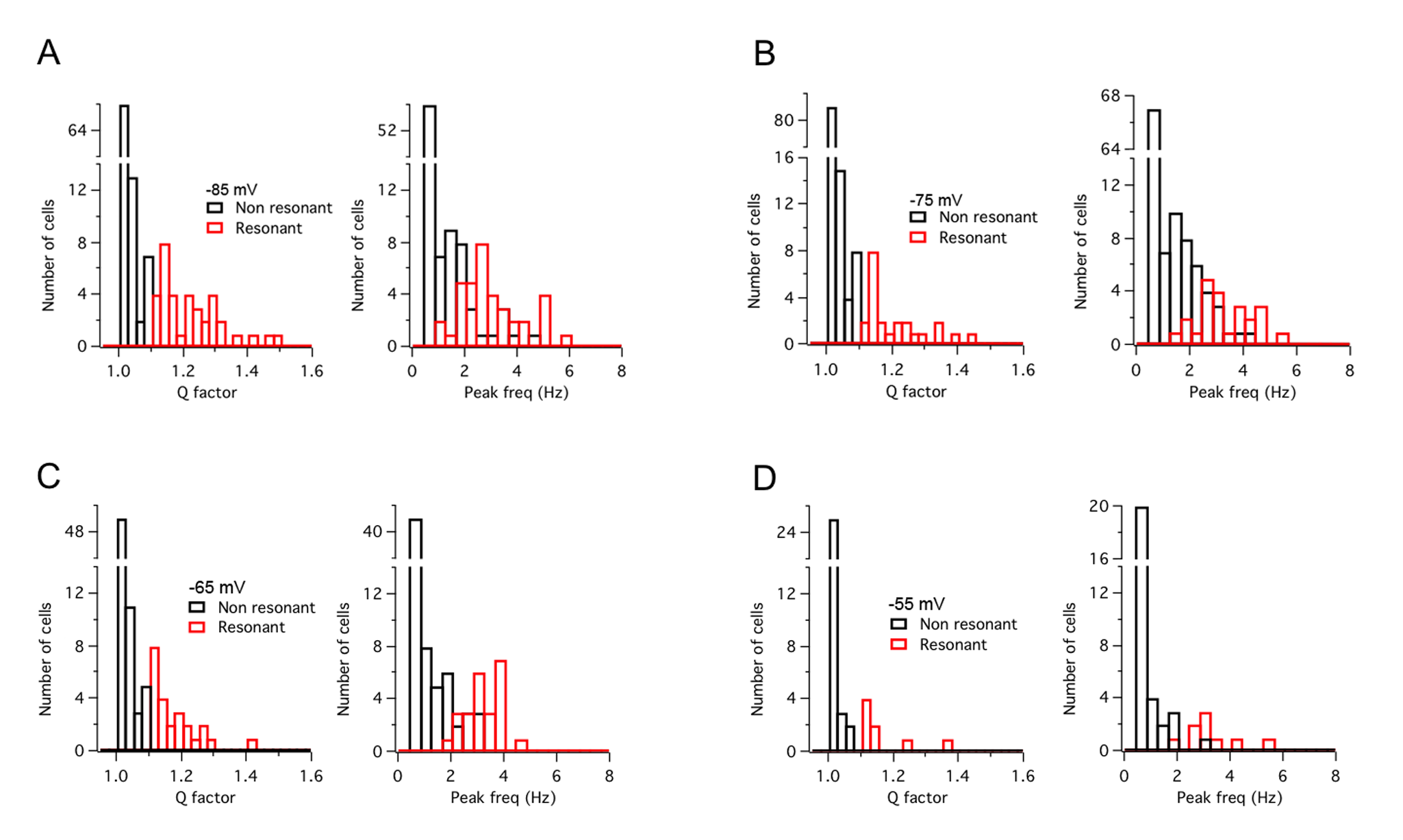

Supplement: Figure S1 — Q value and peak frequency distributions for different voltage ranges. A–D, Histograms of the Q factors (left) and frequencies at which Z reaches its maximum (peak frequency; right), for ZAP stimulation at different membrane potentials binned every 10 mV (averages: −85, −75, −65 and −55 mV). Data with Q<1.10 are shown in black and those with Q≥1.10, in red. The number of recorded cells per voltage range was 125, 132, 92 and 41, for −85, −75, −65 and −55 mV, respectively. (TIF) [file pone.0085826.s001.tif]

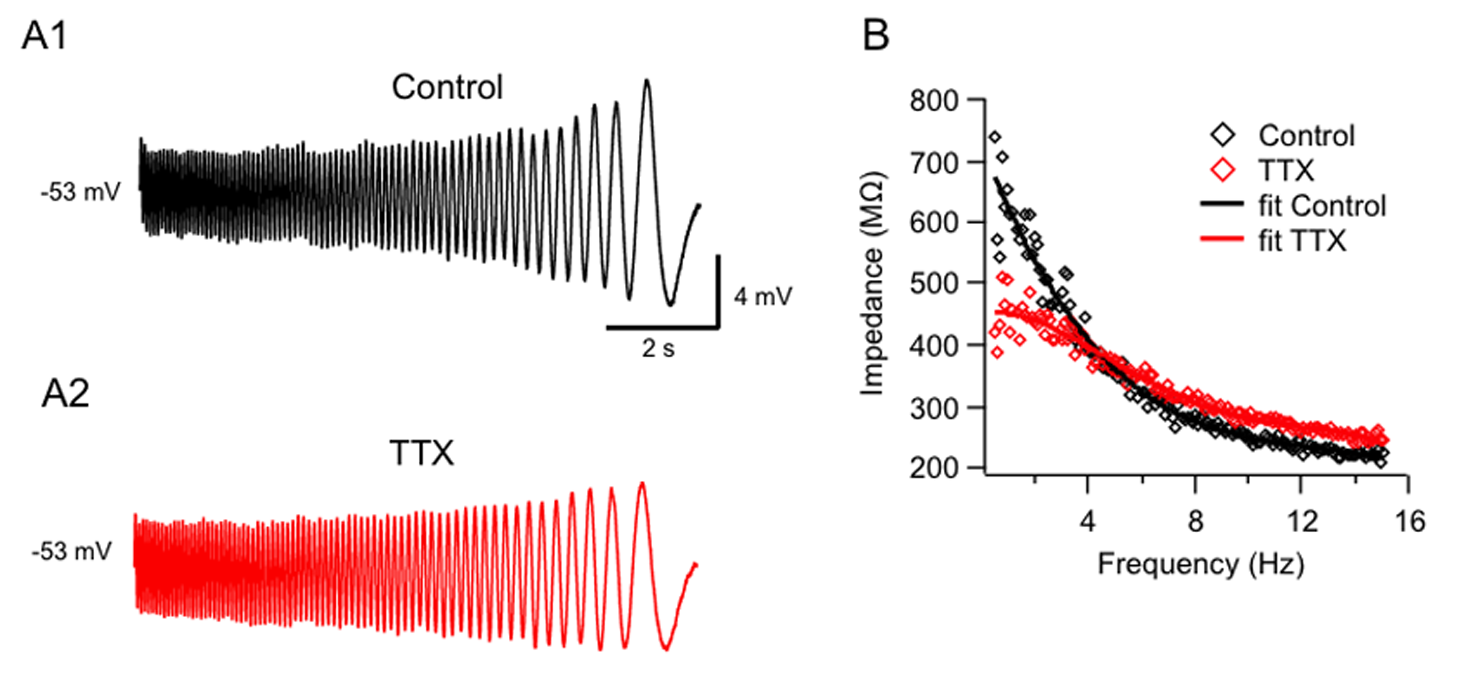

Supplement: Figure S2 — INaP is also present in non-resonant cells. Effect of TTX (1 µM) on the subthreshold responses of a non-resonant neuron. A1, ZAP-stimulation-evoked voltage responses before (Control) and during the extracellular application of TTX (A2). B, Impedance profiles, before and during TTX treatment. (TIF) [file pone.0085826.s002.tif]

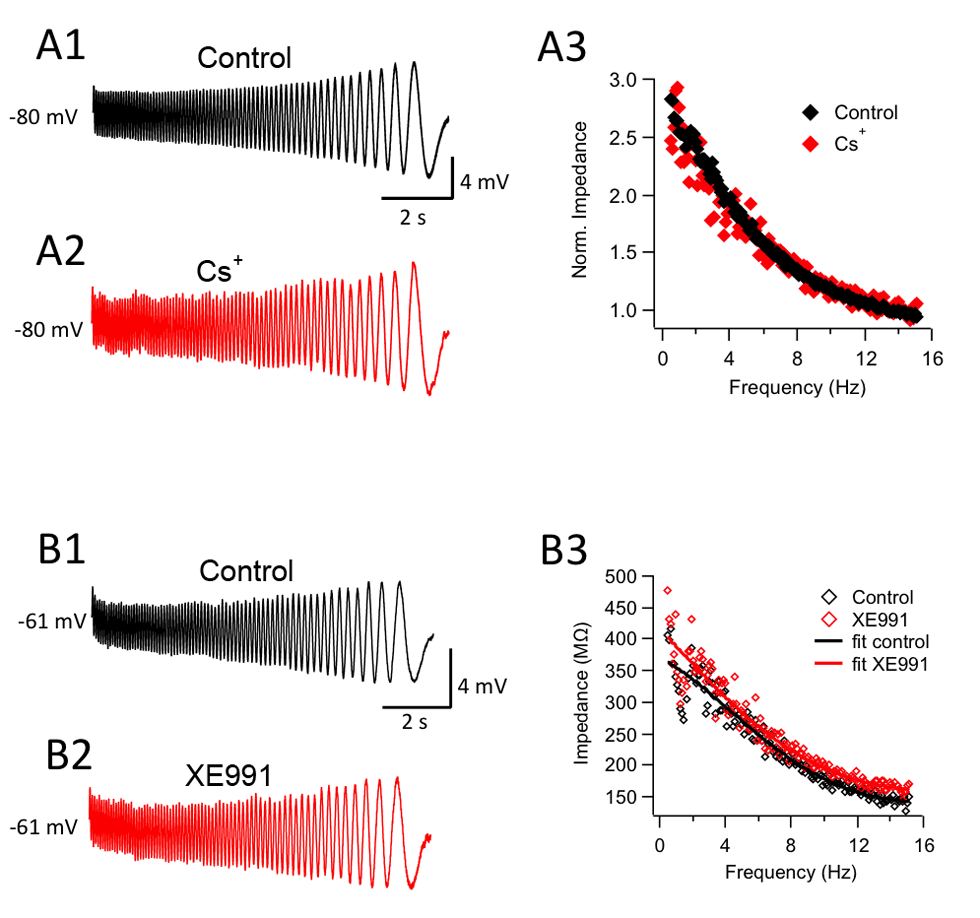

Supplement: Figure S3 — Cs+ and XE991 do not alter impedance profiles of non-resonant neurons. A, ZAP-induced voltage traces at a baseline potential of −80 mV from a non-resonant neuron before (A1) and during the bath application of 4 mM Cs+ (A2). A3, Normalized impedance profiles for the recordings in A1 and A2. B, ZAP-induced voltage traces from a non-resonant neuron before (B1) and during application of 10 µM XE991 (B2) at a baseline membrane potential of −61 mV. B3, Impedance profiles for recordings in B1 and B2. (TIF) [file pone.0085826.s003.tif]

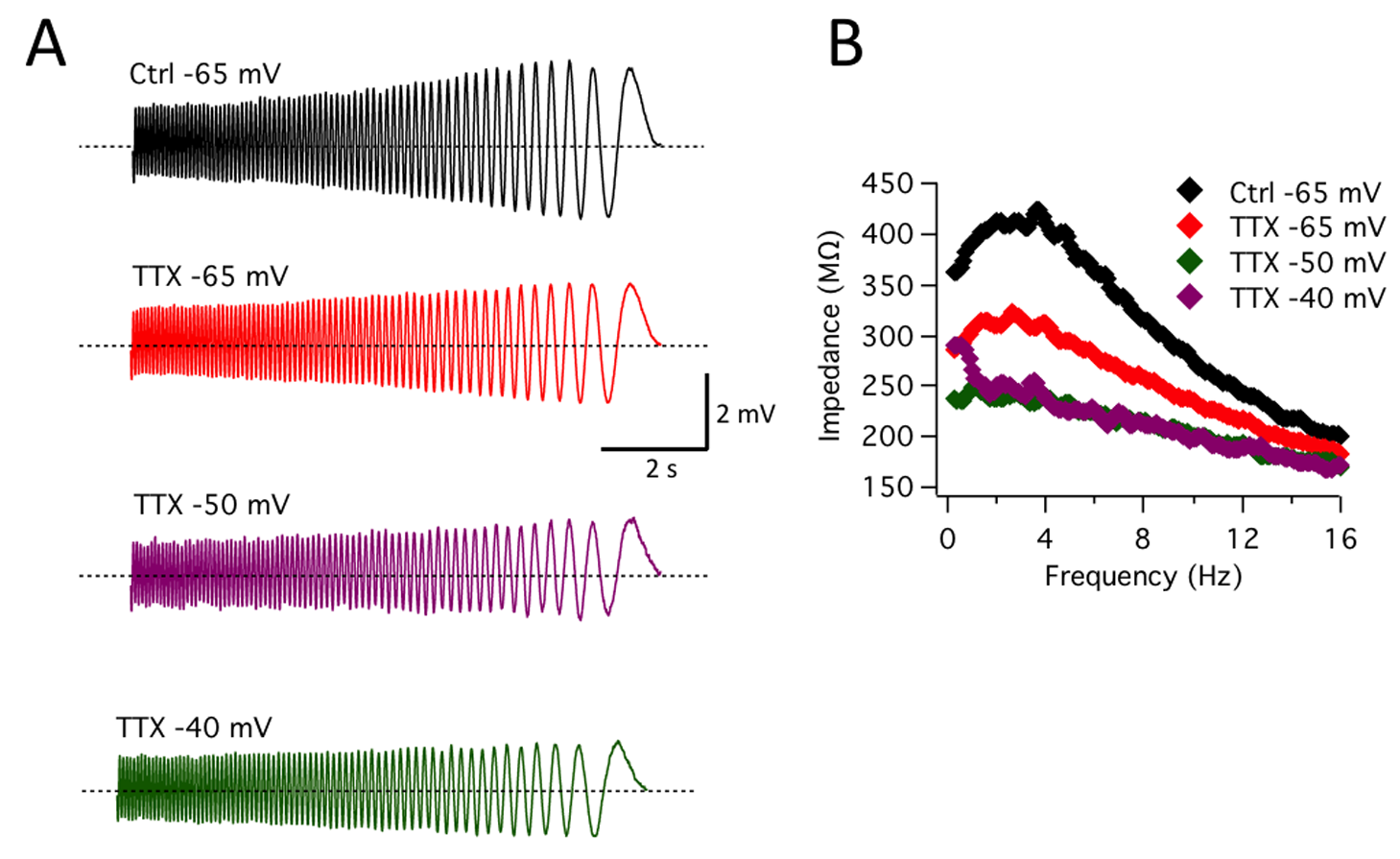

Supplement: Figure S4 — Spike blockade in a neuron resonating at subthreshold voltages revealed the absence of Im -dependent resonance at more depolarized potentials. A, ZAP-induced voltage traces at control conditions and after application of TTX to allow exploration of resonance at suprathreshold potentials. Resonance is observed at −65 mV in both conditions, but it is absent at voltages at which Im is expected to be active and Ih non active (−50 or −40 mV, compare with Figure S6). B, Impedance profiles for traces in A (Q = 1.25 for control and 1.08 in TTX at −65 mV; Q = 1.00 for more depolarized voltages). (TIF) [file pone.0085826.s004.tif]

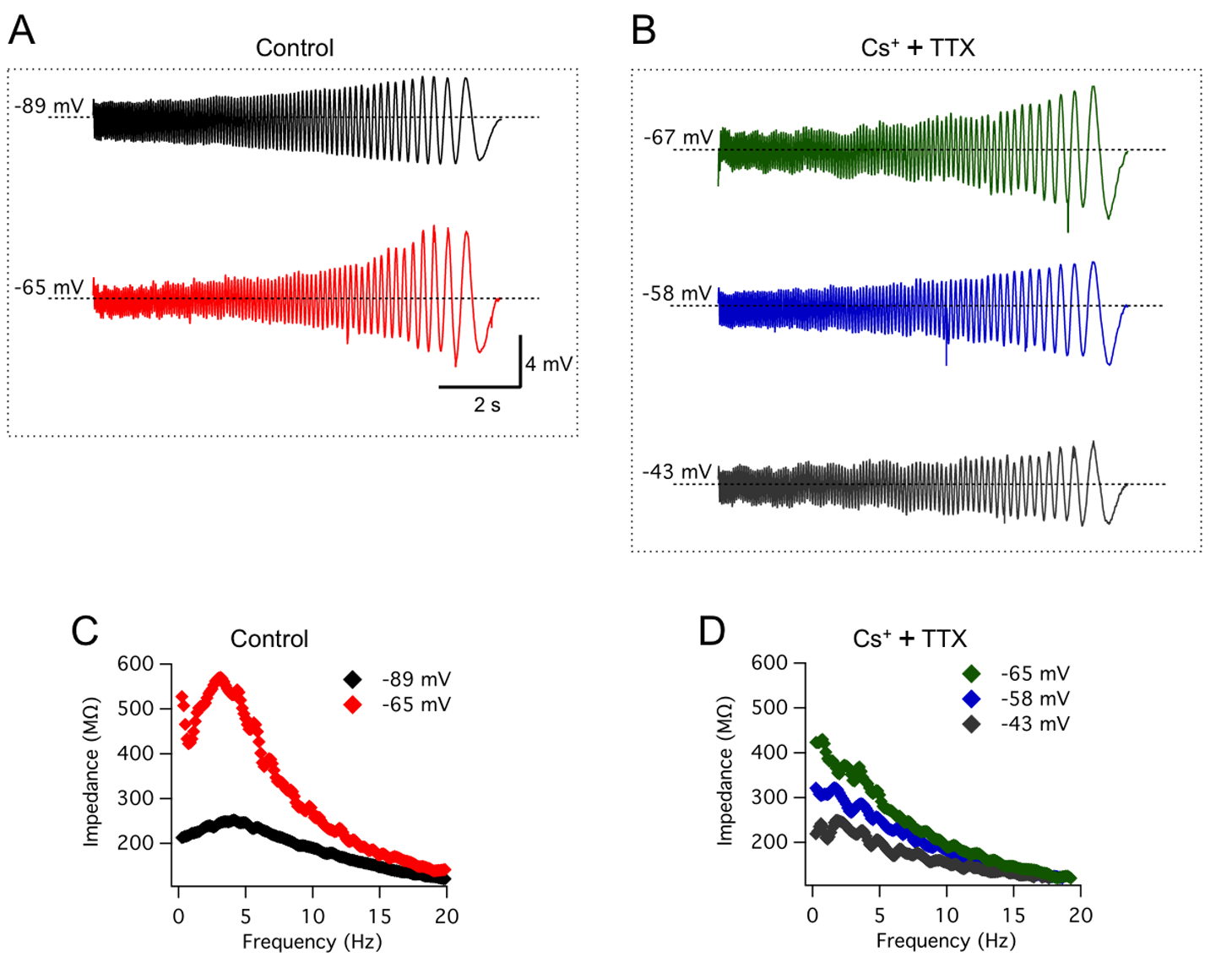

Supplement: Figure S5 — Blockade of Ih -resonance and spikes confirms the lack of the Im -dependent mechanism in another ACo neuron. A, ZAP-induced voltage traces at −89 and −65 mV in control conditions (Q and fres are 1.2 at 3.7 Hz and 1.4 at 2.9 Hz, respectively). B, To evaluate the existence of Im-dependent resonance in this neuron, voltage traces were recorded in the presence of TTX (to block spikes) and Cs+ (4 mM; to eliminate Ih-dependent resonance). Subthreshold resonance was completely eliminated and it is absent even at −43 mV where Im is supposed to be fully active (compare with Figure S6). C and D, impedance profiles for traces in A and B, respectively. (TIF) [file pone.0085826.s005.tif]

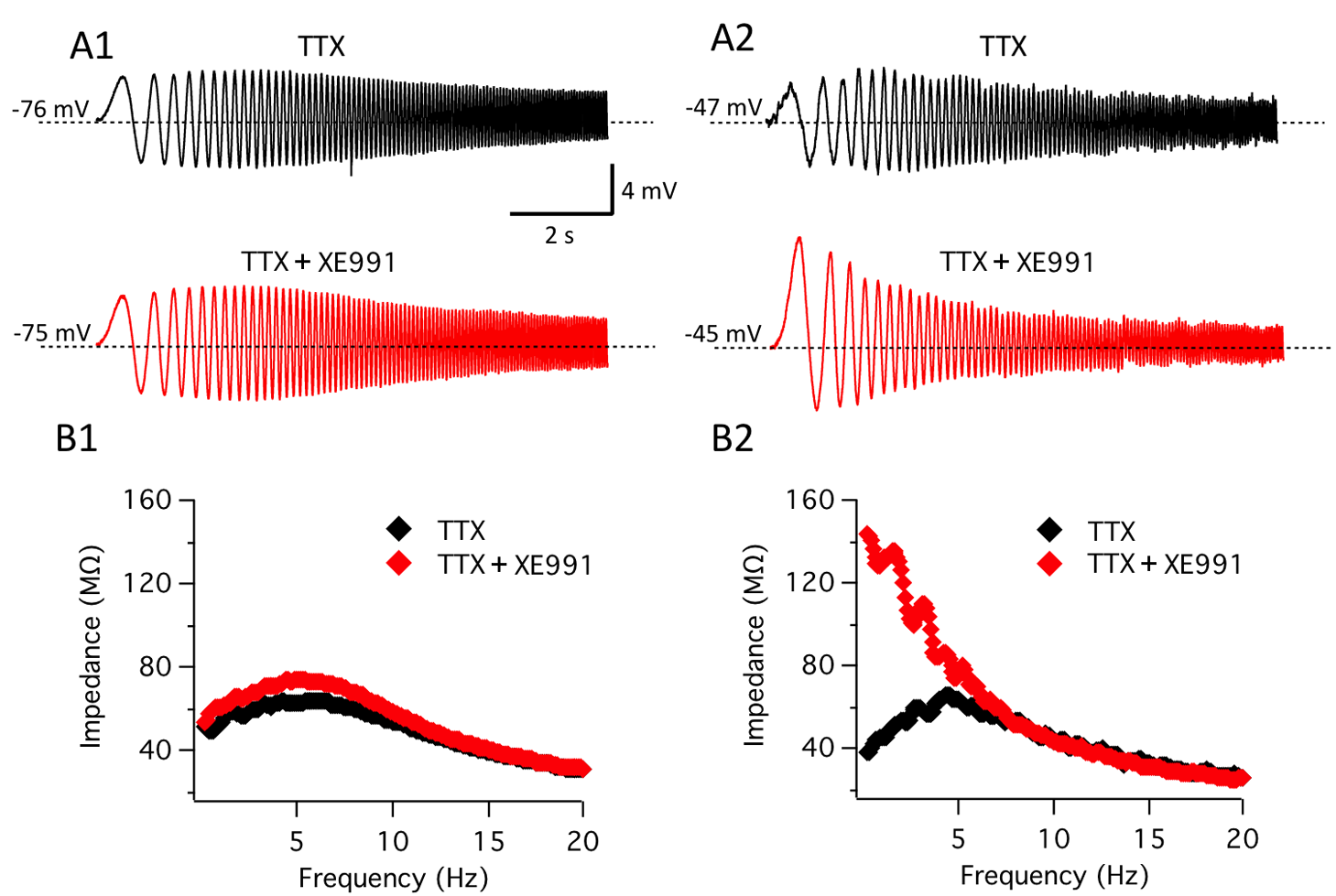

Supplement: Figure S6 — Example of a hippocampal resonant neuron showing a strong Im -dependent resonance at suprathreshold potentials in TTX. A, ZAP-induced voltage traces under TTX and after the application of 10 µM XE991 to block KCNQ channels, at −75 mV (A1) and at −45 mV (A2). B, impedance profiles for the recordings in (A) showing that resonance at this hyperpolarized potential is not affected by XE991 (B1). In contrast, at the suprathreshold potential (B2) application of the KCNQ blocker confirmed that resonance relies completely on Im at this voltage. (TIF) [file pone.0085826.s006.tif]

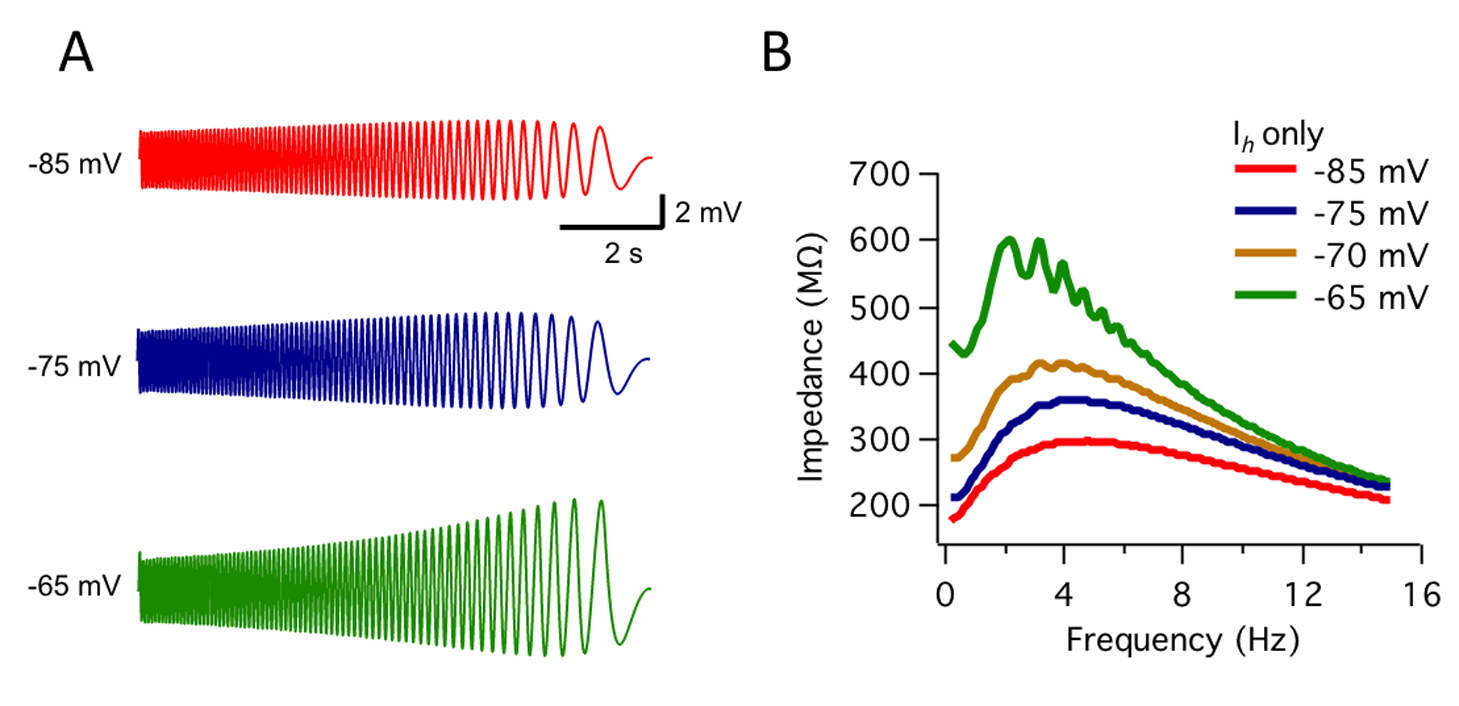

Supplement: Figure S7 — Computer simulations confirm that perithreshold resonance can be generated exclusively by Ih . A, Simulated voltage responses to ZAP stimuli (10 pA, 15-0 Hz, at 30°C) applied at −85, −75 and −65 mV baseline potentials (NEURON 7.0; see Methods). B, Impedance profiles for the traces in A (including also the result for −70 mV). fres and Q values are 4.6 Hz and 1.58 (−85 mV), 4.5 Hz and 1.73 (−75 mV), 3.8 Hz and 1.57 (−70 mV), and 2.8 Hz and 1.4 (−65 mV). Model parameters as shown in Table 3, but with gh = 0.04 mS/cm2 and gm = 0). (TIF) [file pone.0085826.s007.tif]
